# Expression and potential molecular mechanism of TOP2A in metastasis of non-small cell lung cancer

**DOI:** 10.1038/s41598-024-63055-2

**Published:** 2024-05-28

**Authors:** Jiatao Wu, Wenjuan Li, Xueying Zhang, Fan Shi, Qianhao Jia, Yufei Wang, Yuqi Shi, Shiwu Wu, Xiaojing Wang

**Affiliations:** 1 Anhui Province Key Laboratory of Clinical and Preclinical Research in Respiratory Disease, Molecular Diagnosis Center, First Affiliated Hospital, Bengbu Medical University, 287 Changhuai Road, Bengbu, 233004 China; 2 Key Laboratory of Anhui Province Cancer Translational Medicine Center, Bengbu, 233030 China; 3 Department of Pathology, Bengbu Medical University, Bengbu, 233030 China; 4 Anhui No. 2 Provincial People’s Hospital, Hefei, 230041 China

**Keywords:** Non-small cell lung cancer, TOP2A, Wnt/β-catenin signaling pathway, Epithelial–mesenchymal transition, Cell plasticity, Lung cancer, Tumour biomarkers

## Abstract

DNA topoisomerase II alpha (TOP2A) expression, gene alterations, and enzyme activity have been studied in various malignant tumors. Abnormal elevation of TOP2A expression is considered to be related to the development of non-small cell lung cancer (NSCLC). However, its association with tumor metastasis and its mode of action remains unclear. Bioinformatics, real-time quantitative PCR, immunohistochemistry and immunoblotting were used to detect TOP2A expression in NSCLC tissues and cells. Cell migration and invasion assays as well as cytoskeletal staining were performed to analyze the effects of TOP2A on the motility, migration and invasion ability of NSCLC cells. Cell cycle and apoptosis assays were used to verify the effects of TOP2A on apoptosis as well as cycle distribution in NSCLC. TOP2A expression was considerably upregulated in NSCLC and significantly correlated with tumor metastasis and the occurrence of epithelial–mesenchymal transition (EMT) in NSCLC. Additionally, by interacting with the classical ligand Wnt3a, TOP2A may trigger the canonical Wnt signaling pathway in NSCLC. These observations suggest that TOP2A promotes EMT in NSCLC by activating the Wnt/β-catenin signaling pathway and positively regulates malignant events in NSCLC, in addition to its significant association with tumor metastasis. TOP2A promotes the metastasis of NSCLC by stimulating the canonical Wnt signaling pathway and inducing EMT. This study further elucidates the mechanism of action of TOP2A, suggesting that it might be a potential therapeutic target for anti-metastatic therapy.

## Introduction

Lung cancer is the second most prevalent malignant tumor globally, and its incidence and fatality rates are increasing annually^1^. Non-small cell lung cancer (NSCLC) accounts for approximately 85% of the overall incidence of lung cancer^2^. During malignant tumor development, proliferation and invasion are the most crucial factors leading to mortality^2,3^. As the early symptoms of NSCLC are relatively inconspicuous, most patients are diagnosed in the middle and advanced stages. Although there have been significant advances in diagnosing and treating NSCLC in recent years, survival rates have not improved, owing to its recurrence and dissemination. Therefore, it is essential to determine the proteins and regulatory systems responsible for malignant tumor development to increase the percentage of patients with cancer who can overcome the disease.

DNA topoisomerase IIA (TOP2A) plays a significant role in the development, progression, invasion, treatment, and prognosis of cancerous tumors. DNA topoisomerase regulates cell proliferation by participating in cell cycle regulation and apoptosis, and is closely related to tumor chemotherapy drug targets^4,5^. Recent studies have indicated that lung, colon, and pancreatic malignancies are characterized by aberrant expression of tumor TOP2A^6–10^. The aberrant expression of TOP2A is intimately associated with the proliferation, motility, invasion, and epithelial–mesenchymal transition (EMT) events of liver cancer, as well as the poor prognosis of patients with liver cancer, and indicates a possible therapeutic target for liver cancer^11^. The function and mechanism of TOP2A in pancreatic cancer have been fully verified. As a β-catenin co-activator, it activates the actin transduction process and regulates its biological behavior by activating the canonical WNT signaling pathway^7^. Although TOP2A has been demonstrated to play a crucial role in tumors, it is vital to explore how TOP2A promotes metastasis in NSCLC.

EMT is a widespread and complex pathophysiological process that occurs during cancer metastasis. During EMT, normal-epithelial cells miss cell polarity and robust cell–cell junctions, resulting in a shuttle-like interstitial morphology with enhanced motility and mobility^12^. Extensive studies on the function and mechanism of EMT in tumor progression and penetration have revealed that EMT has profound significance in cancer treatment and is a potential target for anti-tumor metastasis therapies^13,14^.

The WNT protein family is essential for genesis, advancement, motility, and distant metastasis. This function may be mediated via the conventional Wnt/β-catenin signaling pathway or β-catenin-independent non-classical pathways^15,16^. As a typical WNT ligand, Wnt3a is inextricably associated with the genesis and pathogenesis of several disorders, including cancer^17^. The classical Wnt signaling cascade leads to cancer by stabilizing β-catenin with Wnt ligands^18,19^. β-catenin has multiple functions, including cell–cell adhesion and signal transduction^20,21^. β-catenin is released from the E-cadherin complex and interacts with other proteins during EMT, unlocking the potential of β-catenin signaling to promote EMT^12,22^.

This study determined whether TOP2A modulates the canonical WNT signaling pathway to impact prognosis, metastatic tumor capability, and EMT occurrence in NSCLC. The results showed that TOP2A is highly expressed in NSCLC, and its expression level is generally associated with poor prognosis and tumor stage in patients with NSCLC. After TOP2A gene silencing in vitro, the proliferation, motility, and invasion of NSCLC cells were drastically diminished. Overexpression of TOP2A significantly promotes migration, invasion, EMT, and cell cycle progression of NSCLC, while significantly inhibiting cell apoptosis. However, when the gene transcription of Wnt3a was silenced, these effects were significantly suppressed.

## Materials and methods

### Clinical specimens and immunohistochemistry

We randomly selected 141 individuals who underwent radical lung cancer surgery to confirm lung squamous cell carcinoma (LUSC) or lung adenocarcinoma (LUAD) using immunohistochemistry. Paraffin sections were prepared from both cancerous and healthy tissues collected from patients. Patients were contacted every 6 months via telephone to obtain a periodic evaluation of their survival status after surgery. All participants provided written informed consent, and the study was approved by the Bengbu Medical College Ethics Committee (No. 2020KY035) and performed according to the principles of the Declaration of Helsinki. The American Joint Commission defines the TNM stage in the Cancer Lung Cancer Staging System, Eighth Edition. Table S1 of Additional File 1 outlines the clinicopathological features of the patients.

Paraffin-embedded tissue samples were sliced into 4 μm-thick sections. After overnight baking, the sections were dewaxed with xylene and dehydrated with gradient alcohol. The slices were treated with a 3% H_2_O_2_ solution for 10–20 min to repair the antigen and to suppress endogenous peroxidase and then rinsed with PBS for 9 min. The appropriate primary antibody was applied to the sections at 4 °C. Subsequently, the sections were incubated for 60 min with a secondary antibody. Before drying in gradient alcohol, the sections were stained using DAB and hematoxylin solutions. β-catenin, E-cadherin, and N-cadherin antibodies were purchased from Fuzhou Maixin Biotech Co., Ltd (Fuzhou, China), whereas TOP2A and Wnt3a antibodies were purchased from Abcam. Two pathologists with extensive experience performed the immunohistochemical evaluation of the sections.

Immunohistochemical evaluation of TOP2A, Wnt3a, β-catenin, E-cadherin, and N-cadherin was based on the percentage of positive cells in the tumor tissue and staining intensity. The scoring rules were as previously described^23^.

### Cell culture

Human NSCLC cell lines (A549, H1299, and PC9) and human normal bronchial epithelial cells (BEAS-2B) were acquired from the ATCC. BEAS-2B was cultured in DMEM containing 10% fetal bovine serum medium (FBS), whereas A549, H1299, and PC9 were grown in RPMI-1640 media supplemented with 10% FBS at 37 °C and 5% CO_2_.

### Plasmid construction, lentiviral construction, and cell transfections

Transfection of small interfering RNAs into NSCLC cells was performed according to the Operation Handbook of the Lipofectamine®3000 reagent (Thermo Fisher Scientific) and it suppressed the production of endogenous TOP2A and Wnt3a when the cell fusion rate reached 50–60%. To establish a stable cell line with elevated TOP2A gene expression, the entire TOP2A gene was cloned into the expression vector Pslenti-EF1-PURO-CMV-3XFlag-WPRE (OBioTechnology, Shanghai, China). The virus was delivered following the manufacturer’s instructions for A549 and H1299 cells. After 48 h, the infected cells were grown in RPMI-1640 medium with puromycin (1 µg/mL) for stable passage. Infected cells were first screened in a medium containing puromycin (2 µg/mL).

### Western blot and quantitative real-time PCR (qRT-PCR)

The proteins of gene-interfered cells were extracted using RIPA buffer. After quantifying the isolated proteins, they were separated on SDS-PAGE and transferred to PVDF membranes. The PVDF membranes were then placed on a shaker and blocked for approximately 100 min at room temperature. Finally, the PVDF membranes were incubated with appropriate primary antibodies at 4 °C overnight. TOP2A and Wnt3a antibodies were manufactured by Abcam, and additional antibodies were manufactured by Boster. The following day, the membrane was washed three times with TBST for 8 min on a shaker, incubated for 1.5 h with secondary antibodies, and photographed using Gel Doc 2000 (Bio-RAD) equipment.

Total RNA was extracted using TRIZOL reagent, and complementary DNA was synthesized using a Reverse Transcription Kit (Takara, Japan). Finally, qRT-PCR was performed using a Roche 480 machine. The results were normalized to β-actin expression levels. The data were processed using the 2^-^^ΔΔCt^ method.

### Transwell assay and staining of the actin cytoskeleton

To perform cell invasion experiment, 100 µL of Matrigel (Corning, USA) was injected into the transwell chamber. After 12 h of incubation in serum-free media, the cells were trypsinized, counted, suspended, and seeded in the upper transwell chamber (Corning, USA). Incubation for 24 or 48 h at 37 °C and 5% CO_2_permitted cells to engage in migratory or invasive motility. The cells were fixed with 4% paraformaldehyde and then stained with crystal violet for 20 min. After staining, five random fields of view (200×) were chosen for counting under the microscope.

Coomassie brilliant blue staining was used to examine the cytoskeleton of the cultured NSCLC cells. Initially, 8** × **10^4^ cells per group were seeded in 6-well plates. The following day, adherent cells were fixed with 4% paraformaldehyde for 20 min, permeabilized with 0.2% Triton-X-100 for 5 min, and then 0.2% Coomassie brilliant blue R-250 was added to 1 mL of solution. Finally, the cells were washed three to five times with PBS, and cytoskeletal alterations were observed using an inverted microscope.

### Cell wound healing assay

Trypsinized cells were seeded at a density of approximately 1** × **10^6^ cells per well in six-well plates and incubated for 24 h (85–95% confluency). Scratch wound healing was photographed at 0, 24, and 48 h after wound creation. In addition, three random fields of view were chosen, cell scratches were recorded using microscopy (4×), and the migratory ability of cells was measured using Image-Pro Plus.

### Analysis of cell apoptosis and cell cycle using flow cytometry

Apoptotic cells were identified using an apoptosis detection kit (Beyotime). First, the cells were digested using trypsin, which did not include EDTA, and then counted. Cells were then incubated with a fluorescent dye solution at room temperature for approximately 15 min in the dark, and the apoptosis rate of cells was detected by flow cytometry.

Before cell cycle analysis, cells were fixed overnight in 75% ethanol at 4 °C. After 16–18 h, the cells were then washed with PBS, and the supernatant was removed after centrifugation. The cells were then incubated with the fluorescent dye PI for 30 min at 37 °C in the dark.

### Statistical analysis

Statistical analysis was performed using GraphPad Prism 9, and all statistical results are presented as mean ± standard deviation (SD). A paired or two independent samples t-test was used to compare two groups, and one-way ANOVA was utilized to analyze multiple groups. A chi-square test was used to analyze the correlation between the expression status of TOP2A and Wnt3a and clinicopathological characteristics. Overall survival (OS) analysis was performed using Kaplan–Meier plots and logarithmic tests. Multivariate survival analysis was performed using Cox proportional hazards analysis. The correlation of TOP2A with WNT3A, CTNNB1, CDH2, MMP2, and MMP9 was tested using Spearman’s correlation coefficient. *P < 0.05, **P < 0.01, ***P < 0.001 and ****P < 0.0001 were considered statistically significant.

### Ethics approval and consent to participate

This study was approved by the Ethics Committee of Bengbu Medical University (No. 2020KY035). All patients provided written informed consent. The study adhered to the ethical guidelines of the Declaration of Helsinki.

## Results

### TOP2A is upregulated in NSCLC

**Figure 1:**
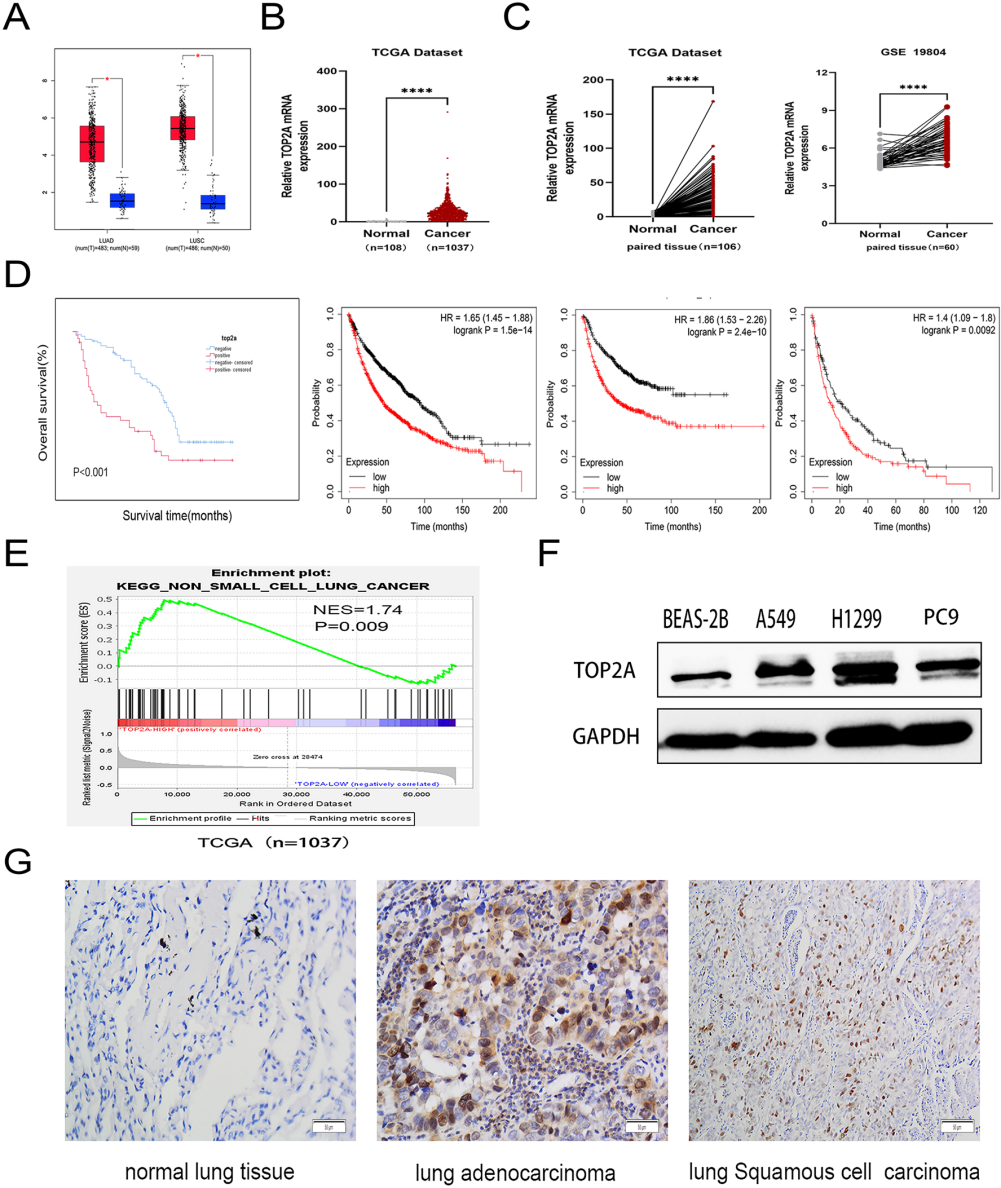
Expression of TOP2A in NSCLC. (**A**) TOP2A was highly expressed in LUAD and LUSC [red bars:tumor tissue; blue bars:normal tissue]. (**B**) TOP2A expression level was higher in NSCLC [Source: TCGA (n = 1037) database]. (**C**) TOP2A expression was notably elevated in NSCLC tissue than in paired normal tissue [Sources: TCGA (n = 108), GSE19804 (n = 60)]. (**D**) Bioinformatics study and validation of clinical specimens revealed that patients with elevated TOP2A levels had a poor prognosis. (**E**) TCGA (n = 1037) data revealed an association between NSCLC and TOP2A expression. (**F**) The levels of TOP2A protein were considerably elevated in NSCLC cells compared to BEAS-2B cells. (**G**) Immunohistochemical analysis demonstrated that TOP2A protein expression was considerably greater in lung cancer tissue than in normal lung tissue (200×). *P < 0.05, **P < 0.01, ***P < 0.001 or ****P < 0.0001. All experiments were repeated three times independently.

The online Gene Expression Profiling Interactive Analysis (GEPIA) tool revealed that TOP2A was ubiquitously expressed in LUAD and LUSC (Fig. 1A). Two relevant microarray gene expression datasets (TCGA and GSE19804) evaluated the NSCLC gene expression profiles. TOP2A expression was considerably higher in NSCLC tissues than in normal lung tissues in both independent and paired samples (P < 0.0001) (Fig. 1B,C). By sorting clinical patient information and performing a Kaplan–Meier survival analysis for patient OS, we determined that patients with increased expression of TOP2A had considerably poor OS. Using the Kaplan–Meier online tool, we subsequently confirmed the association between TOP2A mRNA expression level and the prognosis and survival of patients with NSCLC, and a similar conclusion was reached (Fig. 1D). Further, single-gene enrichment analysis revealed a potential association between TOP2A expression and NSCLC-related genes (NES = 1.74, P = 0.009) (Fig. 1E).

To evaluate whether the expression of TOP2A protein is elevated in NSCLC, the synthesis of TOP2A was evaluated in NSCLC cells and lung cancer tissues. Compared to healthy bronchial epithelial cells, the production of TOP2A protein was dramatically elevated in NSCLC cells (Fig. 1F). In addition, the expression of TOP2A in LUAD and LUSC was found to be significantly higher than that in equivalent normal lung tissue during the validation of clinical tissue samples (Fig. 1G).

### TOP2A promotes NSCLC cell metastasis

**Figure 2:**
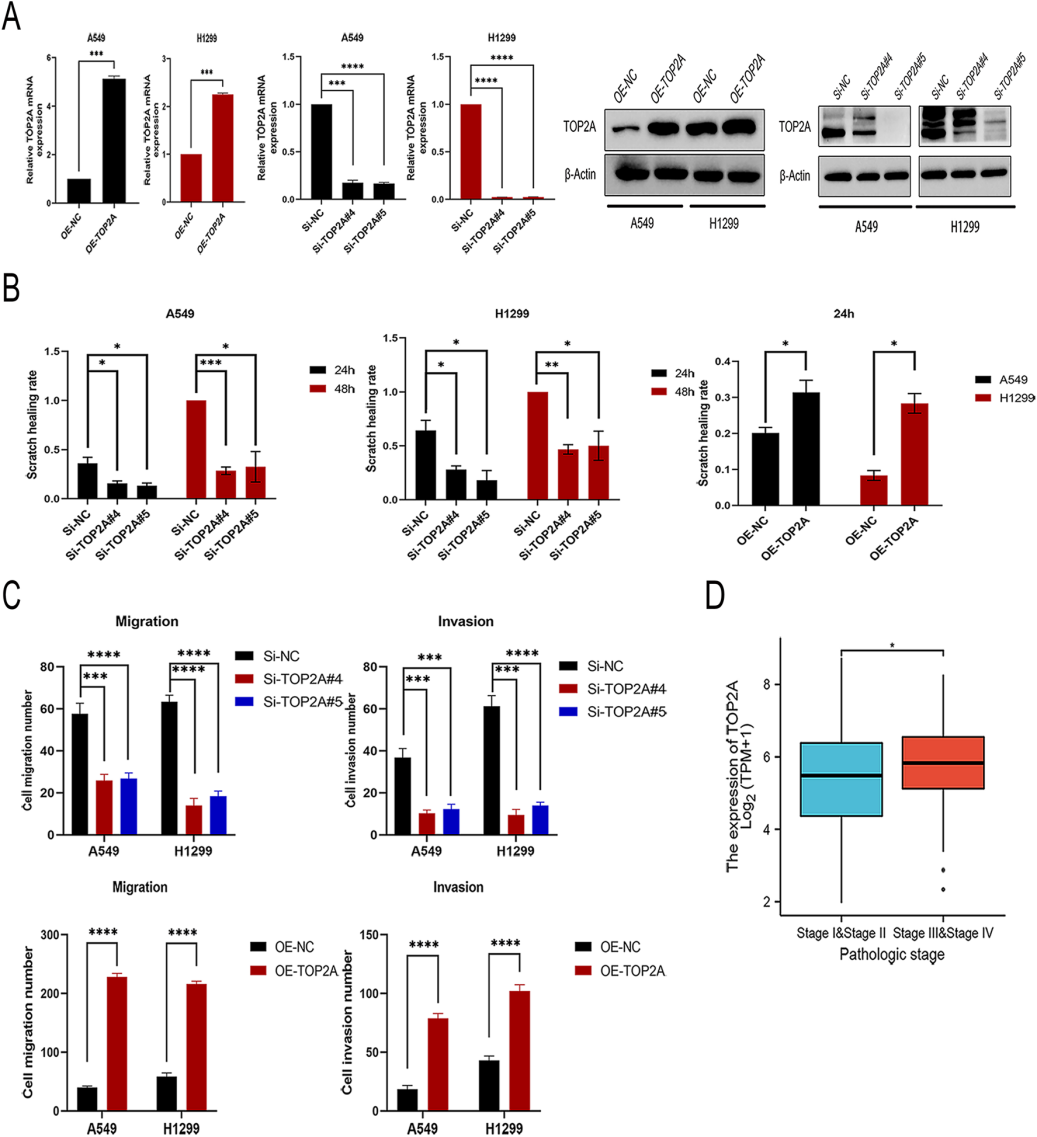
Effect of TOP2A on progression and metastasis of NSCLC. (**A**) A549 and H1299 cells were engineered to produce TOP2A-expressing and knockdown cell lines. (**B**) Wound healing experiment to evaluate the migratory capability of cells overexpressing or silencing TOP2A. (**C**) Transwell assays evaluated migratory and infiltration capacities of cells with TOP2A overexpression or silencing. (**D**) The correlation between TOP2A expression and tumor stage was analyzed based on TCGA data set. *P < 0.05, **P < 0.01, or ***P < 0.001. All experiments were repeated three times independently.

To explore the biological functions of TOP2A in vitro and in vivo, we established NSCLC cell lines that stably and robustly expressed TOP2A by transducing A549 and H1299 cells with lentiviral vectors containing the full-length TOP2A gene. TOP2A-knockdown NSCLC cell lines were constructed using SiRNAs (SiTOP2A). The levels of TOP2A protein were considerably higher in A549-TOP2A and H1299-TOP2A cells than in the negative control group, 48 h after transfection. However, TOP2A expression was considerably lower in A549-SiTOP2A#4, A549-SiTOP2A#5, H1299-SiTOP2A#4, and H1299-SiTOP2A#5 cells (Fig. 2A).

The wound-healing assay indicated that A549-TOP2A and H1299-TOP2A cells exhibited faster wound healing, whereas A549-SiTOP2A#4, A549-SiTOP2A#5, H1299-SiTOP2A#4, and H1299-SiTOP2A#5 cells exhibited comparatively slower wound healing (Fig. 2B, Additional File 4: Fig. S1A). In addition, results from transwell assay showed that overexpression of TOP2A significantly increases NSCLC cell motility and invasion. The converse was true in TOP2A downregulated cells, i.e., TOP2A downregulation significantly inhibited the mobility and infiltration of NSCLC cells (Fig. 2C, Additional File 4: Fig. S1B).

In addition, we investigated the correlation between the transcriptional level of TOP2A and tumor stage using TCGA database (Fig. 2D) and reached a consistent conclusion. Finally, OS-based univariate and multivariate analyses of the pathological features of clinical patients revealed that TOP2A was an independent risk factor influencing the prognosis of NSCLC (Additional File 2, Table S2).

### TOP2A promotes EMT

**Figure 3:**
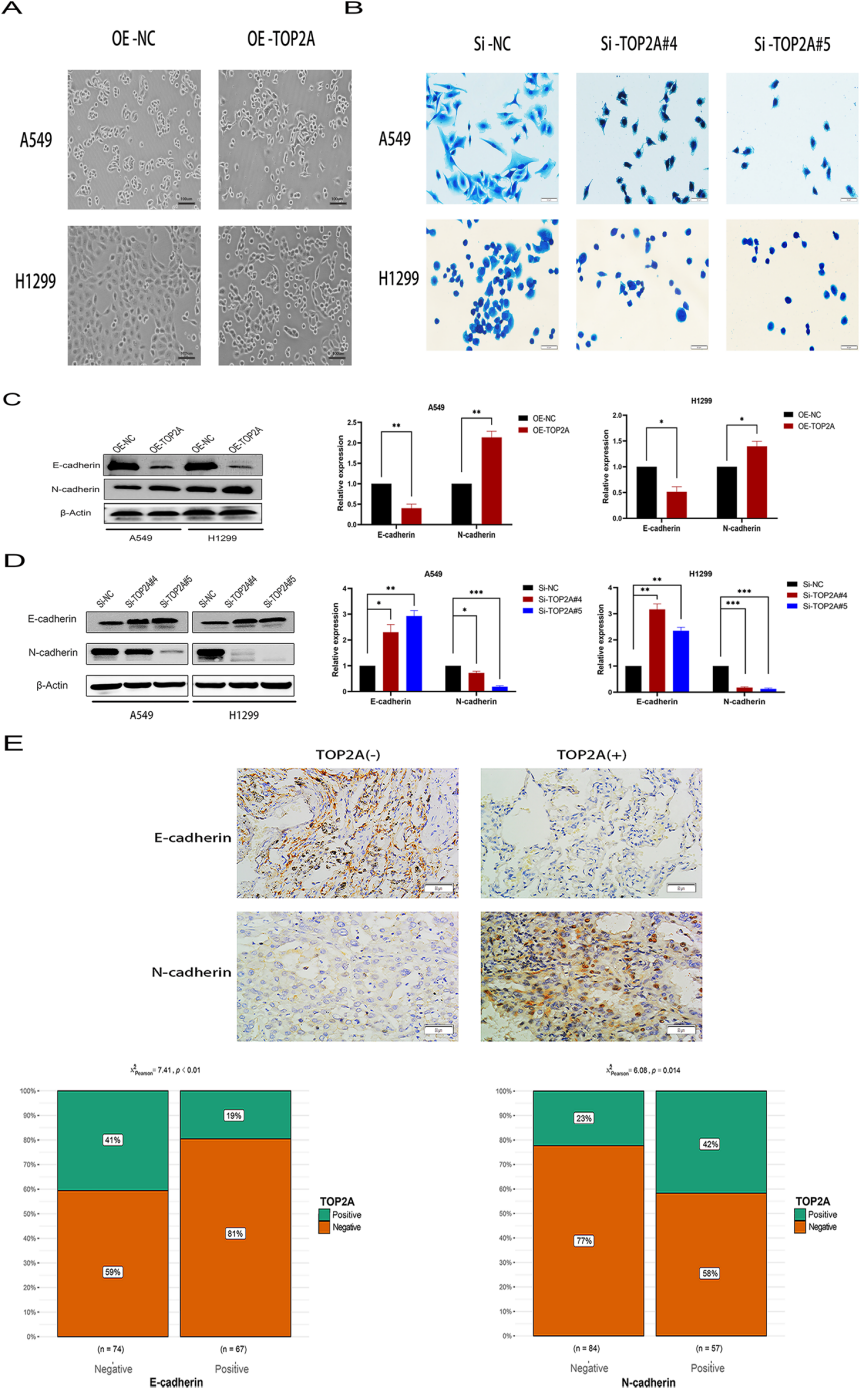
TOP2A promotes progression and metastasis of NSCLC by EMT. (**A**) Under an inverted microscope, the cell morphology in each group was investigated (100×). (**B**) The effect of TOP2A gene knockdown on cytoskeleton was evaluated via the Coomassie brilliant blue staining. (**C,D**) The levels of EMT-related proteins were quantified in the overexpression and knockdown groups of TOP2A. (**E**) Immunohistochemistry was used to evaluate E-cadherin and N-cadherin levels in TOP2A-positive and TOP2A-negative groups (200×). *P < 0.05, **P < 0.01, or ***P < 0.001. All experiments were repeated three times independently.

First, cell morphology observation showed that when TOP2A was overexpressed, the morphology of A549 and H1299 cells changed to a shuttle shape (Fig. 3A). Subsequent Coomassie brilliant blue staining showed that A549 and H1299 cells exhibited shrinkage and disorder of stress fibers after TOP2A knockdown (Fig. 3B). Changing cell morphology to elongated spindle-shaped cells and loss of cell–cell tight junctions characterize the EMT-associated morphological alterations in cells^13,24^. Using the GEPIA website, we noted that TOP2A mRNA was significantly associated with EMT-related molecules such as N-cadherin (CDH2) (Additional File 5: Fig. S2A). Using the GEO dataset, we performed a molecular correlation analysis. We identified a statistically significant association between TOP2A mRNA expression and CDH2 and MMP9 expression (Additional File 5: Fig. S2B). Furthermore, MMP2 and MMP9 mRNA levels were elevated in NSCLC cell lines overexpressing TOP2A (Additional File 5: Fig. S2C).

To determine whether TOP2A expression affected the incidence of EMT, we examined the expression of EMT-related proteins in the overexpression and knockdown states of TOP2A. E-cadherin expression was considerably downregulated in A549-TOP2A and H1299-TOP2A cells relative to the control group but was upregulated in A549-SiTOP2A#4, A549-SiTOP2A#5, H1299-SiTOP2A#4, and H1299-SiTOP2A#5 cells. In addition, we observed that N-cadherin expression was upregulated in A549-TOP2A and H1299-TOP2A cells. However, N-cadherin expression was suppressed in A549-SiTOP2A#4, A549-SiTOP2A#5, H1299-SiTOP2A#4, and H1299-SiTOP2A#5 cells (Fig. 3C,D). E-cadherin expression in patients with lung cancer was negatively correlated with TOP2A, but N-cadherin expression was positively correlated with TOP2A, indicating that TOP2A may facilitate EMT (Fig. 3E). These results demonstrate that TOP2A augments the metastatic potential of NSCLC cells.

### TOP2A stimulates Wnt/β-catenin signal transduction in NSCLC

**Figure 4:**
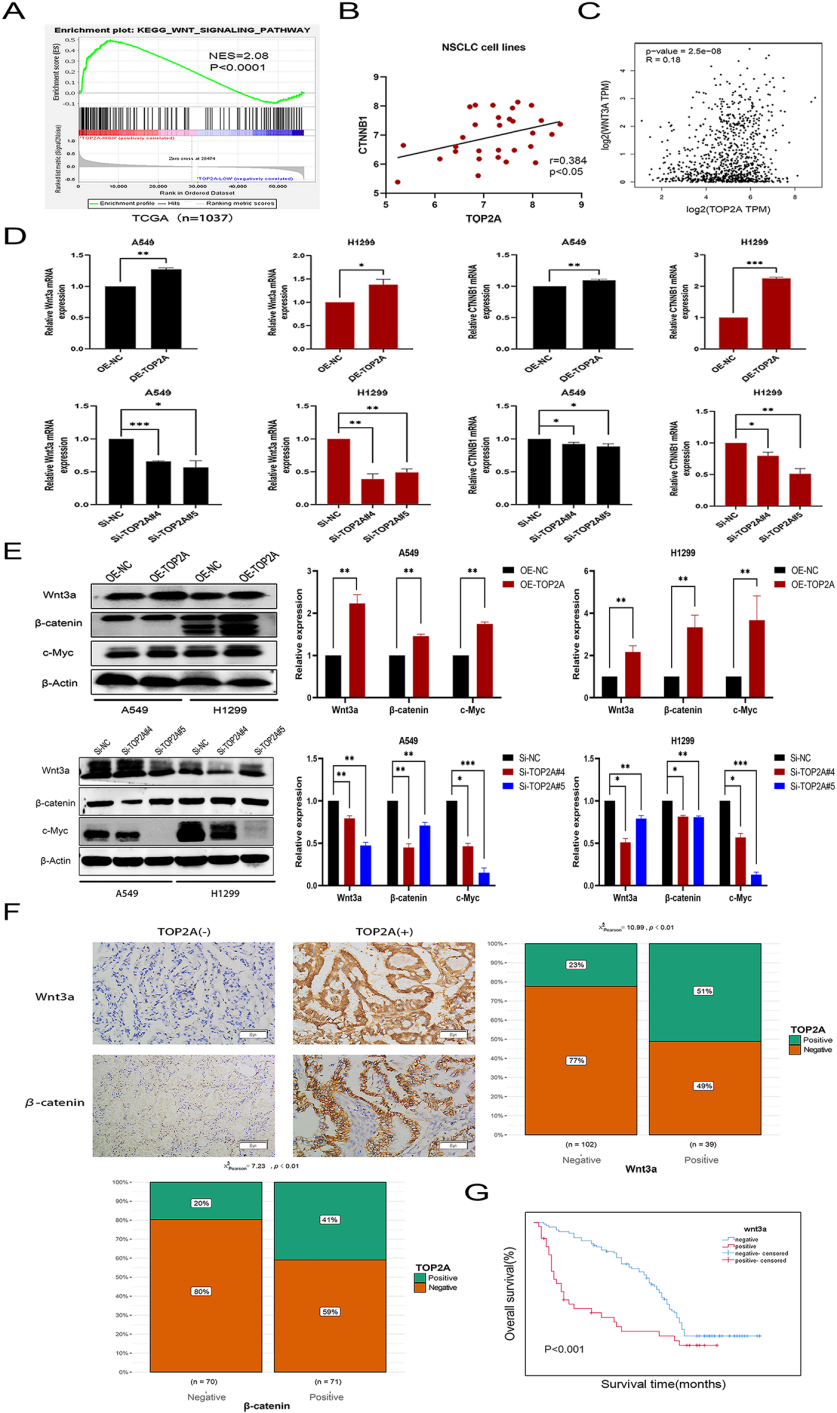
TOP2A is involved in the progression and development of NSCLC via the Wnt/β-catenin signaling cascade. (**A**) TCGA enrichment analysis revealed that TOP2A expression is enriched in the Wnt signaling pathway (n = 1037). (**B**) Several NSCLC cell lines exhibited a clear association between the transcription of TOP2A and β-catenin. (**C**) The online GEPIA exhibited a correlation between TOP2A and WNT3A gene expression levels. (**D,E**) TOP2A overexpression and knockdown groups were examined for gene and protein levels of TOP2A, Wnt3a, and β-catenin. (**F**) Immunohistochemical assessment of Wnt3a and β-catenin expression in the TOP2A-positive and TOP2A-negative groups (200×). (**G**) Patients with high Wnt3a overexpression had an abysmal survival rate. *P < 0.05, **P < 0.01, or ***P < 0.001. All experiments were repeated three times independently.

GSEA analysis based on TCGA and GSE19804 datasets revealed a significant association between the level of TOP2A and the Wnt signaling pathway (Fig. 4A). Using the CCLE database, we identified a significant association between the mRNA levels of TOP2A and human β-catenin, CTNNB1 in NSCLC cell lines (Fig. 4B). Using the online program GEPIA, we performed a correlation analysis of the genes associated with the WNT pathway. Multiple molecules (WNT3A, MYC, TCF3 and LEF1) of the Wnt/β-catenin signal transduction pathway were recognized as statistically significant positive regulators of TOP2A expression (Fig. 4C, Additional File 5: Fig. S2D). We validated the synthesis of Wnt3a, β-catenin, and c-Myc in TOP2A overexpressing or knockdown cells to verify the findings of the bioinformatics analysis. The final experimental findings demonstrated that Wnt3a, β-catenin, and c-Myc expression levels were elevated when TOP2A was overexpressed and diminished in response to TOP2A knockdown (Fig. 4D,E).

Immunohistochemical analysis of human tissue samples indicated a significant association between TOP2A, Wnt3a, and β-catenin expression (Fig. 4F). Furthermore, when correlated with clinicopathological characteristics, Wnt3a expression level was substantially associated with the patient’s prognosis, tumor stage, and lymph node metastasis (Fig. 4G, Additional File 3: Table S3). These findings indicate that TOP2A may activate the Wnt signaling pathway by interacting with the classical ligand Wnt3a, boosting tumor formation, development, and metastasis.

### TOP2A targets Wnt3a, and Wnt3a knockdown suppresses Wnt/β-catenin signaling

**Figure 5:**
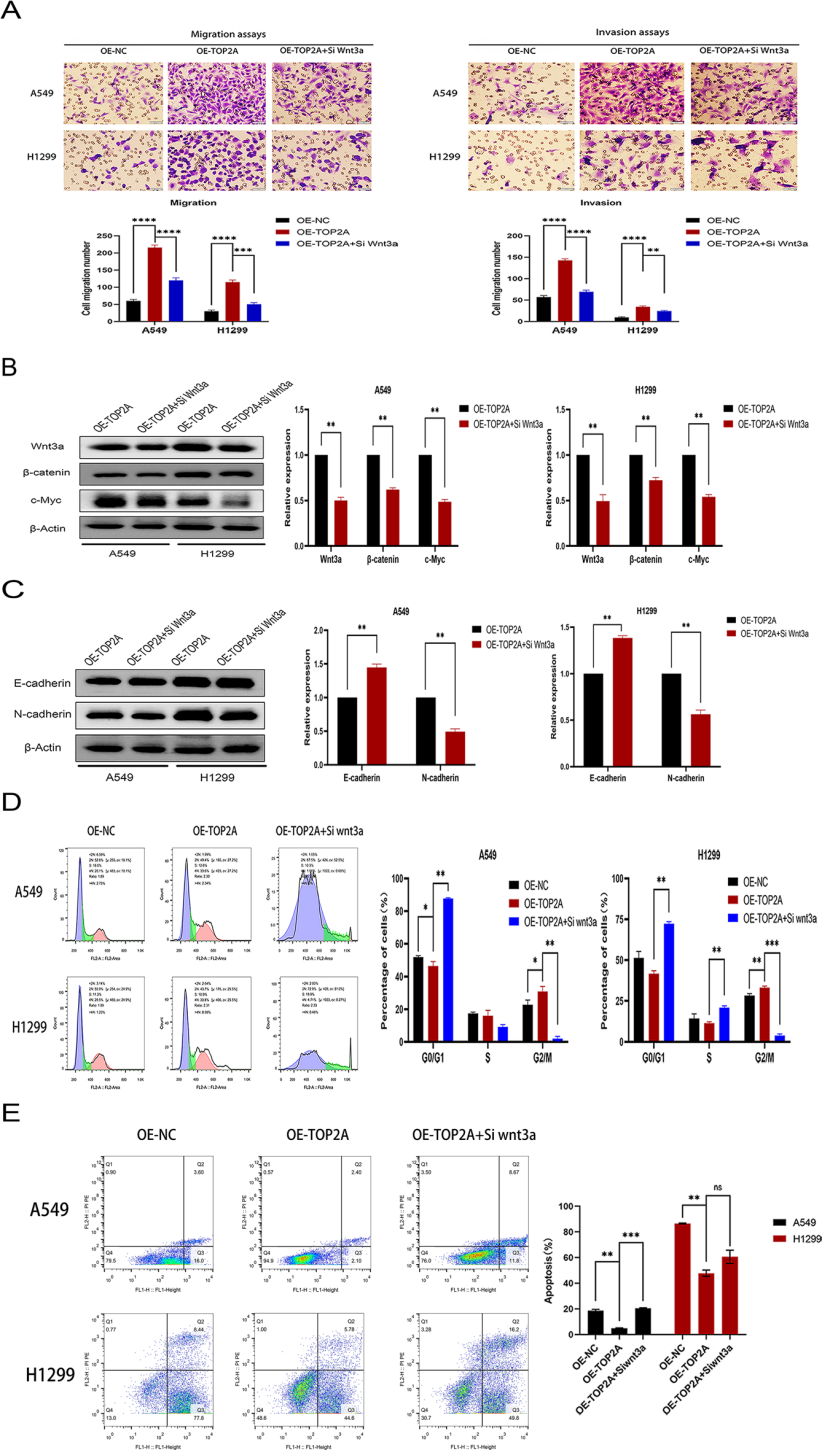
TOP2A promotes non-small cell lung cancer progression via Wnt/β-catenin signaling pathway. (**A**) After Wnt3a gene knockdown, the mobility and invading capabilities of TOP2A-overexpressing cells were evaluated using the transwell assay. (**B,C**) Expression of components of Wnt/β-catenin signaling pathway and EMT-related proteins was examined using western blotting. (**D**) Flow cytometry was utilized to examine the cell cycle. (**E**) Analysis of apoptosis via flow cytometry. *P < 0.05, **P < 0.01, ***P < 0.001 or ****P < 0.0001; *ns:* not signiffcant.

First, qRT-PCR was used to select small interfering RNAs with the highest knockdown efficiency for Wnt3a. When NSCLC cells were treated with Wnt3a-targeting siRNA, their invasiveness and ability to metastasize were dramatically reduced (Fig. 5A). When Wnt3a gene expression was suppressed, the synthesis of β-catenin, a crucial protein in the Wnt signaling cascade, and c-Myc, a downstream target gene protein, was diminished (Fig. 5B). In addition, Wnt3a knockdown inhibited TOP2A-induced changes in the expression of EMT-related molecules (Fig. 5C).

When Wnt3a was knocked down by targeted siRNA, the cell cycle was severely halted in the G0/G1 phase compared to the TOP2A overexpression group (Fig. 5D). Moreover, the absence of Wnt3a significantly increased the number of apoptotic cells (Fig. 5E).

## Discussion

The role of TOP2A has been studied to varying degrees in different cancer types, and it is overexpressed in a variety of solid tumors, particularly during tumorigenesis^6,24–26^. Some studies on pancreatic cancer have shown that TOP2A modulates its biological behavior by activating the canonical Wnt signaling pathway and acting as a co-activator of β-catenin to stimulate actin transduction. miR-144-3p functions in liver cancer cell proliferation, motility, infiltration, and EMT pathways by modulating TOP2A, which may be considered a potential target for liver anticancer therapy^7–9^. According to studies on the etiology of gastric cancer, FAM230B suppresses the transcription of miR-27a-5p, and miR-27a-5p modulates the biological behavior of gastric cancer by diminishing the synthesis of TOP2A^27^. TOP2A has similar functions in breast cancer^28^.

Although a preliminary association between TOP2A and the incidence or progression of NSCLC has been demonstrated, the precise mechanism by which TOP2A promotes tumor metastasis in NSCLC remains unclear. The present study investigated the function and mechanism of TOP2A activation in NSCLC metastasis. By evaluating TOP2A expression levels in matched cells and tissues, we found that TOP2A was dramatically elevated in NSCLC. Next, we determined that TOP2A acts as an oncoprotein that promotes NSCLC metastasis, and the downregulation of its expression has the opposite effect. In addition, we discovered that TOP2A might enhance EMT in NSCLC cells. Mechanistically, TOP2A may activate the Wnt signaling pathway by interacting with the canonical ligand Wnt3a to stimulate β-catenin entry into the nucleus, thereby promoting NSCLC metastasis.

EMT is indispensable in cancer cell migration, invasion, and progression. During this period, epithelial cells transform into mesenchymal cells, E-cadherin expression diminishes, and N-cadherin expression improves^12,13,29,30^. To determine the function and specific mechanism of TOP2A in NSCLC metastasis, we further investigated the association between TOP2A and EMT. Similar to cell migration and invasion assay results, TOP2A upregulation expedited the EMT process in NSCLC cells, wherein E-cadherin expression was diminished and N-cadherin expression continued to increase and induced cell contraction and the emergence of stress fibers. The Wnt signaling pathway is crucial for controlling the EMT process and is involved in several malignancies^31–34^. β-catenin promotes EMT by binding to the TCF/LEF transcription factor family members in the nucleus. In addition, recent studies have shown that MMPs are regulated by the WNT signaling pathway^35–38^, and MMP-2 and MMP-9 can be used as interstitial markers of the EMT process. Consistent with TCGA and GEO datasets, this study revealed increased TOP2A expression in NSCLC. Furthermore, TOP2A expression was strongly associated with NSCLC metastasis, and it modulated the EMT process in NSCLC. It downregulated E-cadherin synthesis and simultaneously upregulated the production of N-cadherin, MMP-2, and MMP-9, consequently promoting cell motility and infiltration. In conclusion, our results demonstrated that TOP2A accelerates cancer progression in NSCLC, and this impact is intimately associated with the EMT process.

Several studies have demonstrated that increased TOP2A expression in glioma cells is strongly associated with tumor metastasis and reduced survival in patients with glioma and that TOP2A is a prominent Wnt pathway activator in glioma, boosting cell growth, motility, and penetration^25,39^. Other studies also demonstrated a link between TOP2A and the Wnt signaling pathway^7,9^. Using bioinformatics and biological experiments, we identified that the expression of TOP2A was strongly associated with Wnt signaling pathway stimulation. Furthermore, when TOP2A was ubiquitously expressed or inhibited, the levels of crucial molecule β-catenin and the target protein c-Myc of the canonical Wnt signaling pathway increased and decrease, respectively.

We noted that when Wnt3a expression was suppressed in A549-TOP2A and H1299-TOP2A cells, TOP2A protein expression decreased, and cell migration and penetration were significantly inhibited. These results suggest that the canonical Wnt signaling pathway may control TOP2A expression in a feedback manner. However, the role of TOP2A in NSCLC metastasis, the specific signaling pathway, and this feedback mechanism have not been further validated in corresponding animal models.

In conclusion, this study reveals that the oncogene TOP2A modulates the canonical Wnt signaling pathway by interacting with Wnt3a and consequently increasing metastasis and EMT processes in patients with NSCLC. These results expand our knowledge by providing a mechanistic basis for NSCLC metastasis. In addition, the Wnt/β-catenin pathway exerts a positive feedback on TOP2A expression. Further investigation is required to explore the potential for TOP2A as a crucial biomarker for the prognosis and survival of patients with NSCLC as well as an effective anti-metastatic therapy target.

## Conclusions

The results of this study demonstrate that TOP2A plays an essential role in NSCLC tumor dissemination by stimulating the Wnt/β-catenin signaling pathway and EMT process. Therefore, TOP2A may be a suitable therapeutic target for anti-metastatic therapy.

### Supplementary Information

Supplementary Tables.

Supplementary Figures.

Supplementary Legends.

Supplementary Information.

## Data Availability

All data generated or analysed during this study are included in this manuscript and its Supplementary Information files.
