# Supplementary material for: Expression and potential molecular mechanism of TOP2A in metastasis of non-small cell lung cancer
Source: Sci Rep. 2024 May 28;14:12228. doi: 10.1038/s41598-024-63055-2 (PMC11133405; doi:10.1038/s41598-024-63055-2)
Supplement: Supplementary file 1 — Supplementary Tables. [file 41598_2024_63055_MOESM1_ESM.pdf]

Table S1. Patient characteristics.

Characteristic	Frequency (n)	Percentage (%)
Age (years)		
<60	65	46.1
≥60	76	53.9
Gender		
Female	61	43.3
Male	80	56.7
Smoking		
No	93	66
Yes	48	34
Gross Type		
Central	72	51.1
Peripheral	69	48.9
Histologic Type		
SCC	21	14.9
Ade	120	85.1
Grade		
Well	30	21.3
Moderate	81	57.4
Poor	30	21.3
LNM		
No	80	56.7
Yes	61	43.3
TNM stage		
I	61	43.3
II	35	24.8
III	45	31.9

Abbreviations: SCC, squamous cell carcinoma; Ade, adenocarcinoma; LNM, lymph node metastasis; TNM, tumor-node-metastasis.

Table S2. Univariate and multivariate analysis of the OS and clinicopathological variables.

		Univariable Analysis		Multivariable Analysis	
Variables	Number	HR (95% CI)	P	HR (95% CI)	P
TOP2A		2.303 (1.514–3.502)	<0.001	2.469 (1.470–4.146)	0.001
Negative	98				
Positive	43				
wnt3a		1.997 (1.292–3.086)	0.002	0.550 (0.314–0.964)	0.037
Negative	102				
Positive	39				
Age (years)		0.936 (0.632–1.388)	0.744		
<60	65				
≥60	76				
Gender		0.711 (0.477–1.058)	0.093		
Female	61				
Male	80				
Smoking		0.768 (0.500–1.180)	0.229		
No	93				
Yes	48				
Gross Type		0.888 (0.600–1.316)	0.555		
Central	72				
Peripheral	69				
Histologic Type		1.619 (0.947–2.770)	0.078		
SCC	21				
Ade	120				
Grade		2.017 (1.470–2.768)	<0.001	2.005 (1.423–2.826)	<0.001
Well	30				
Moderate	81				
Poor	30				
LNM		3.185 (2.130–4.763)	<0.001	4.151 (2.534–6.800)	<0.001
No	80				
Yes	61				
TNM stage		2.230 (1.753–2.838)	<0.001	2.124 (1.636–2.758)	<0.001
I	61				
II	35				
III	45				

Abbreviations: SCC, squamous cell carcinoma; Ade, adenocarcinoma; LNM, lymph node metastasis; TNM, tumor-node-metastasis.

Table S3. The correlation between TOP2A or Wnt3a and the clinical pathological characteristics in NSCLC.

Variables	TOP2a		P	wnt3a		P
	Negative	Positive		Negative	Positive	
Age (years)			0.161			0.712
＜60	49	16		48	17	
⩾60	49	27		54	22	
Gender			0.001			0.141
Female	51	10		48	13	
Male	47	33		54	26	
Smoking			<0.001			0.279
No	75	18		70	23	
Yes	23	25		32	16	
Gross Type			0.065			0.683
Central	45	27		51	21	
Peripheral	53	16		51	18	
Histologic Type			0.018			0.247
SCC	10	11		13	8	
Ade	88	32		89	31	
Grade			0.199			0.008
Well	23	7		24	6	
Moderate	58	23		63	18	
Poor	17	13		15	15	
LNM			0.606			<0.001
No	57	23		69	11	
Yes	41	20		33	28	
TNM stage			0.223			<0.001
I	47	14		55	6	
II	23	12		22	13	
III	28	17		25	20	

Abbreviations: SCC, squamous cell carcinoma; Ade, adenocarcinoma; LNM, lymph node metastasis; TNM, tumor-node-metastasis.

P-values were determined using Pearson's chi-squared analysis.
