# Supplementary material for: Expression and potential molecular mechanism of TOP2A in metastasis of non-small cell lung cancer
Source: Sci Rep. 2024 May 28;14:12228. doi: 10.1038/s41598-024-63055-2 (PMC11133405; doi:10.1038/s41598-024-63055-2)
Supplement: Supplementary file 2 — Supplementary Figures. [file 41598_2024_63055_MOESM2_ESM.pdf]

Figure S1

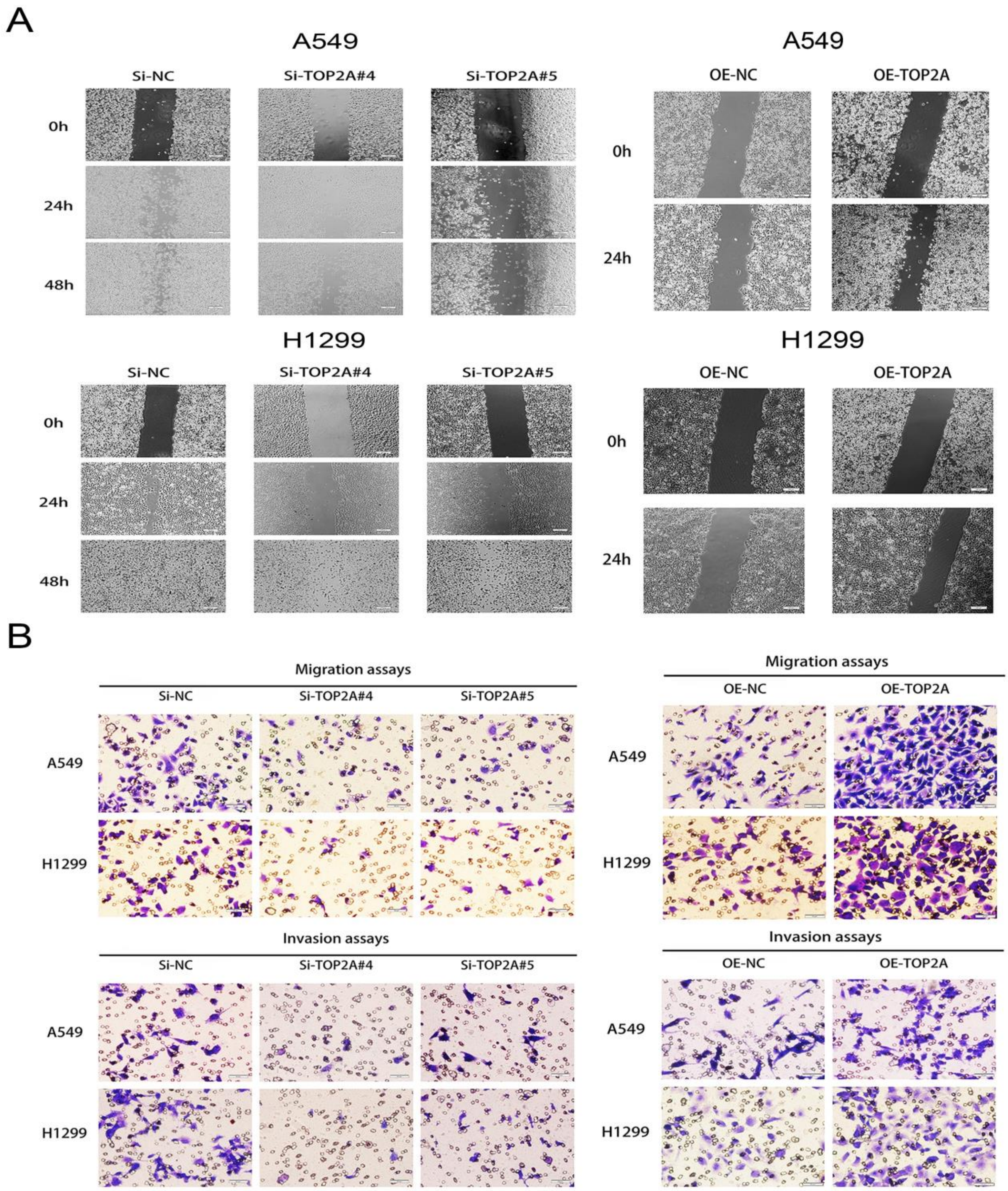

Figure S1: Experimental results of scratching, migration and invasion of A549 and H1299 cells after experimental treatment. (A) The migratory

ability of cells in overexpression and knockdown groups of TOP2A was determined using the wound healing assay. (B) Transwell assay was used to evaluate the motility and invasive ability of cells in which TOP2A was overexpressed, or TOP2A expression was inhibited.

Figure S2

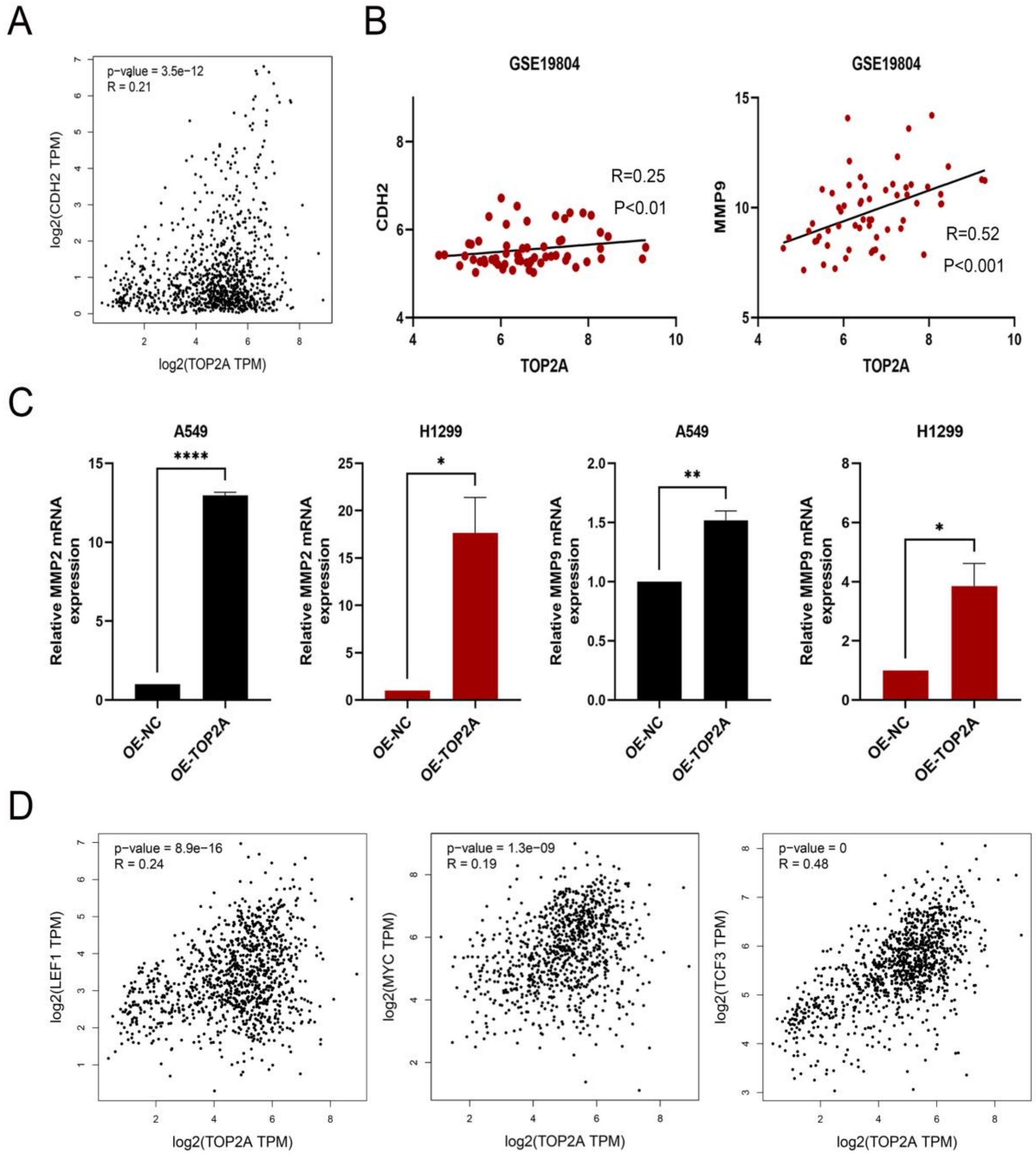

Figure S2: Correlation of TOP2A with EMT-related molecules and core canonical WNT pathway molecules. (A) A significant association between TOP2A and EMT target gene expression. (B) A strong relationship between TOP2A expression and the mRNA levels of CDH2 and MMP9. (C) The transcription of MMP2 and MMP9 was considerably elevated in TOP2A-overexpressing cells compared to the cells of the control group. (D) The GEPIA online tool showed that TOP2A expression was significantly associated with LEF1, TCF3 and MYC.
