# Supplementary material for: Expression and potential molecular mechanism of TOP2A in metastasis of non-small cell lung cancer
Source: Sci Rep. 2024 May 28;14:12228. doi: 10.1038/s41598-024-63055-2 (PMC11133405; doi:10.1038/s41598-024-63055-2)
Supplement: Supplementary file 3 — Supplementary Legends. [file 41598_2024_63055_MOESM3_ESM.docx]

**Expression and potential molecular mechanism of TOP2A in metastasis of non-small cell lung cancer**

Jiatao Wu,^1^ Wenjuan Li,^1^ Xueying Zhang,^1^ Fan Shi,^3^ Qianhao Jia,^3^ Yufei Wang,^3^ Yuqi Shi,^2^ Shiwu Wu,^2,3,4*^ and Xiaojing Wang^1*^

^1^ Anhui Province Key Laboratory of Clinical and Preclinical Research in Respiratory Disease，Molecular Diagnosis Center，First Affiliated Hospital，Bengbu Medical University, 287 Changhuai Road, Bengbu 233004.

^2^Key Laboratory of Anhui Province Cancer Translational Medicine Center,233030, Bengbu,China.

^3^Department of Pathology, Bengbu Medical University,233030, Bengbu,China.

^4^Anhui No. 2 Provincial People’s Hospital, Hefei 230041, China.

Correspondence should be addressed to Shiwu Wu and Xiaojing Wang; wushiwu@bbmc.edu.cn and wangxiaojing8888@163.com.

*Corresponding authors:

**Shiwu Wu:** Key Laboratory of Anhui Province Cancer Translational Medicine Center,233030, Bengbu,China; Department of Pathology, Bengbu Medical University,233030, Bengbu,China; Anhui No. 2 Provincial People’s Hospital, Hefei 230041, China.

**Xiaojing Wang:** Anhui Province Key Laboratory of Clinical and Preclinical Research in Respiratory Disease，Molecular Diagnosis Center，First Affiliated Hospital，Bengbu Medical University, 287 Changhuai Road, Bengbu 233004.

**Supplementary Table S1:** Patients characteristics.

**Supplementary Table S2:** Univariate and multivariate analysis of OS and clinicopathological variables.

**Supplementary Table S3:** The correlation between TOP2A or Wnt3a and clinicopathological characteristics in NSCLC.

**Supplementary Original western blot images:** Original western blot images of Figure1F; Figure2A; Figure3C; Figure3D; Figure4E; Figure5B; Figure5C in the manuscript.

**Supplementary Figure S1**

Experimental results of scratching, migration and invasion of A549 and H1299 cells after experimental treatment. (A) The migratory ability of cells in overexpression and knockdown groups of TOP2A was determined using the wound healing assay. (B) Transwell assay was used to evaluate the motility and invasive ability of cells in which TOP2A was overexpressed, or TOP2A expression was inhibited.

**Supplementary** **Figure S2**

Correlation of TOP2A with EMT-related molecules and core canonical WNT pathway molecules. (A) A significant association between TOP2A and EMT target gene expression. (B) A strong relationship between TOP2A expression and the mRNA levels of CDH2 and MMP9. (C) The transcription of MMP2 and MMP9 was considerably elevated in TOP2A-overexpressing cells compared to the cells of the control group. (D) The GEPIA online tool showed that TOP2A expression was significantly associated with LEF1, TCF3 and MYC.
