# Supplementary material for: Expression and potential molecular mechanism of TOP2A in metastasis of non-small cell lung cancer
Source: Sci Rep. 2024 May 28;14:12228. doi: 10.1038/s41598-024-63055-2 (PMC11133405; doi:10.1038/s41598-024-63055-2)
Supplement: Supplementary file 4 — Supplementary Information. [file 41598_2024_63055_MOESM4_ESM.pdf]

Figure1 F

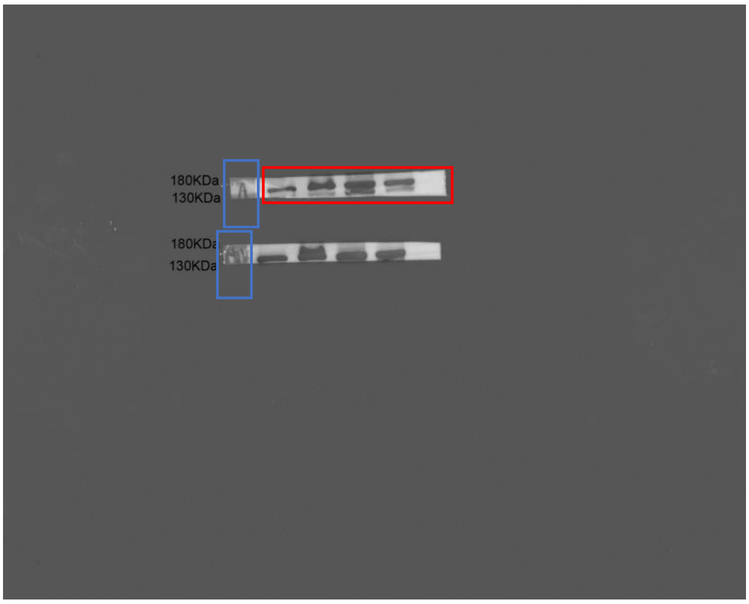

Original western blot image of TOP2A; this band was partially cleaved for the detection of other proteins before hybridization with the antibody.

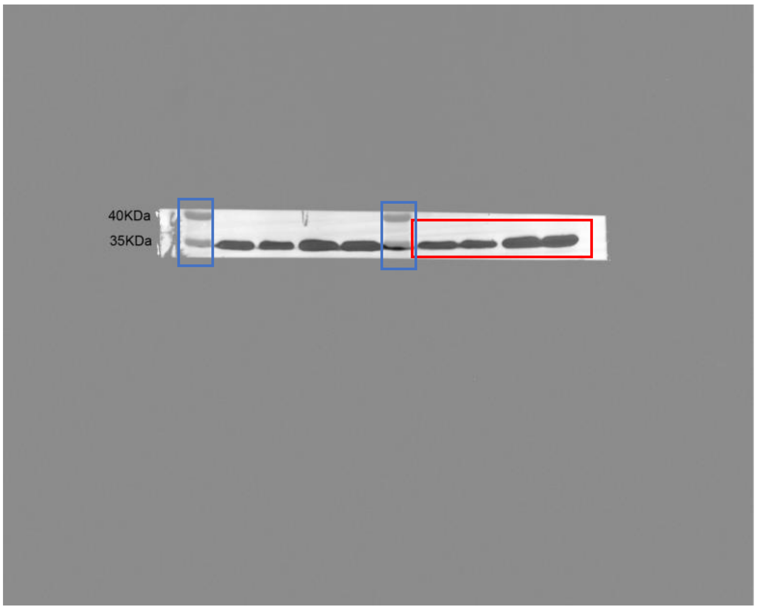

Original western blot image of GAPDH (TOP2A) ; this band was partially cleaved for the detection of other proteins before hybridization with the antibody.

Figure2 A

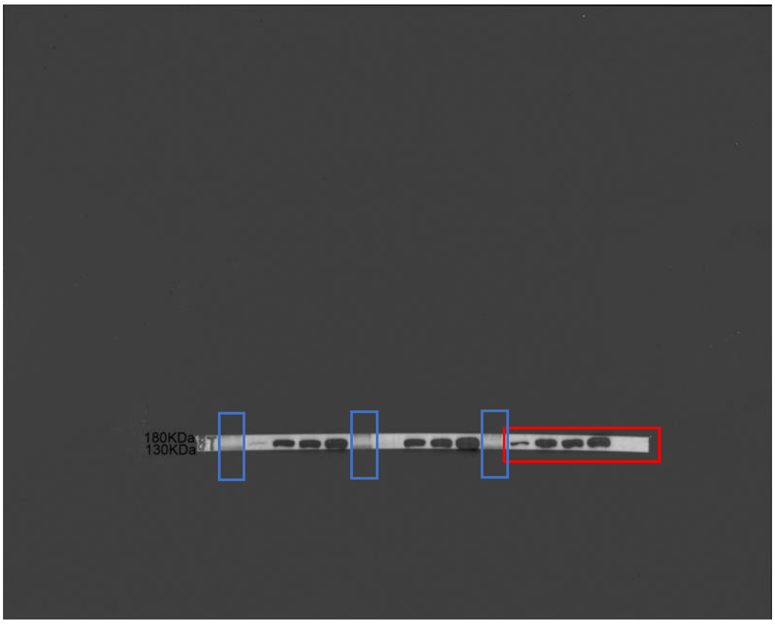

Original western blot image of TOP2A-OE.

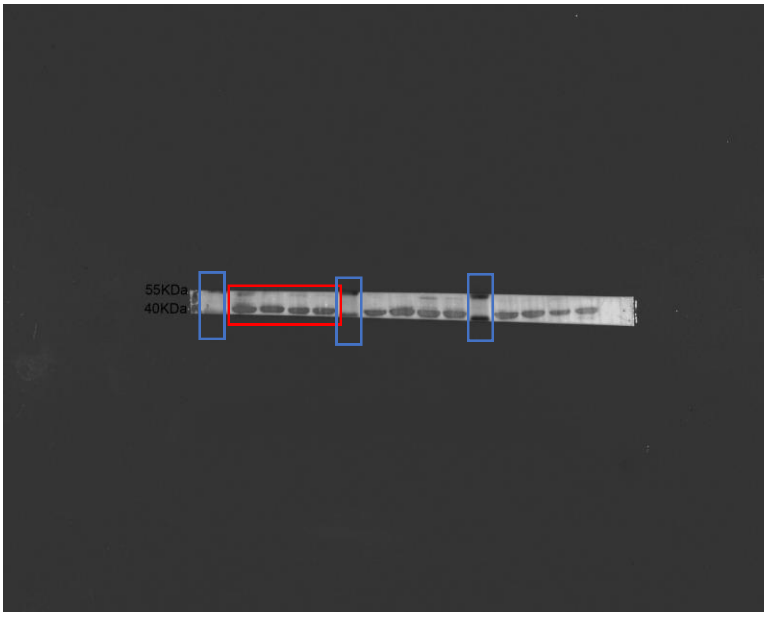

Original western blot image of β-actin(TOP2A-OE).

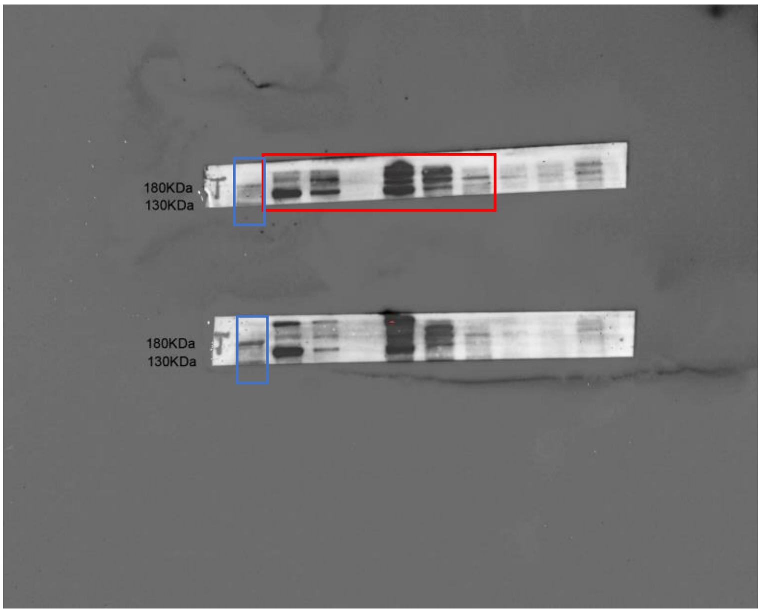

Original western blot image of TOP2A-SiRNA; this band was partially cleaved for the detection of other proteins before hybridization with the antibody.

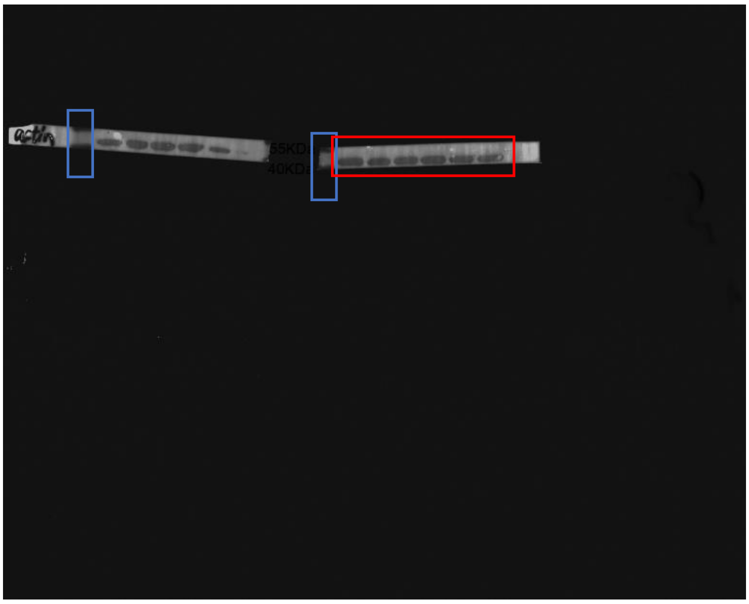

Original western blot image of β-actin(TOP2A- SiRNA) ; this band was partially cleaved for the detection of other proteins before hybridization with the antibody.

Figure3 C

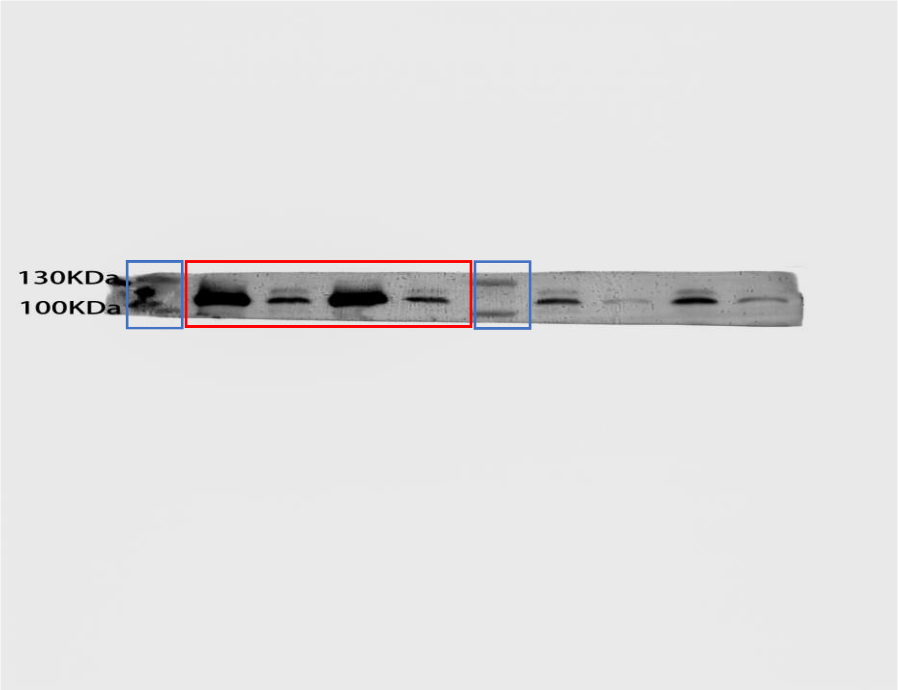

Original western blot image of E-cadherin; this band was partially cleaved for the detection of other proteins before hybridization with the antibody.

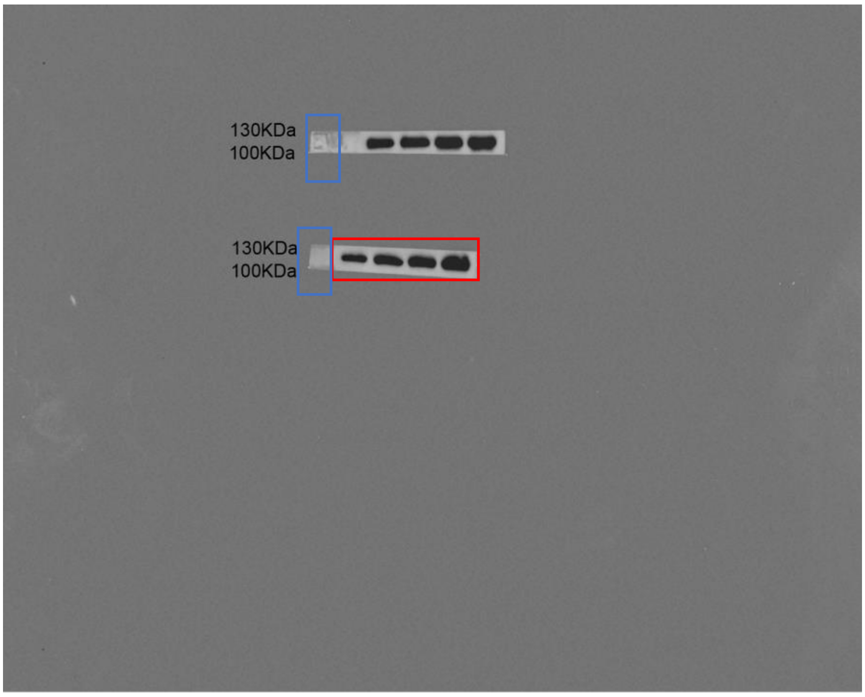

Original western blot image of N-cadherin; this band was partially cleaved for the detection of other proteins before hybridization with the antibody.

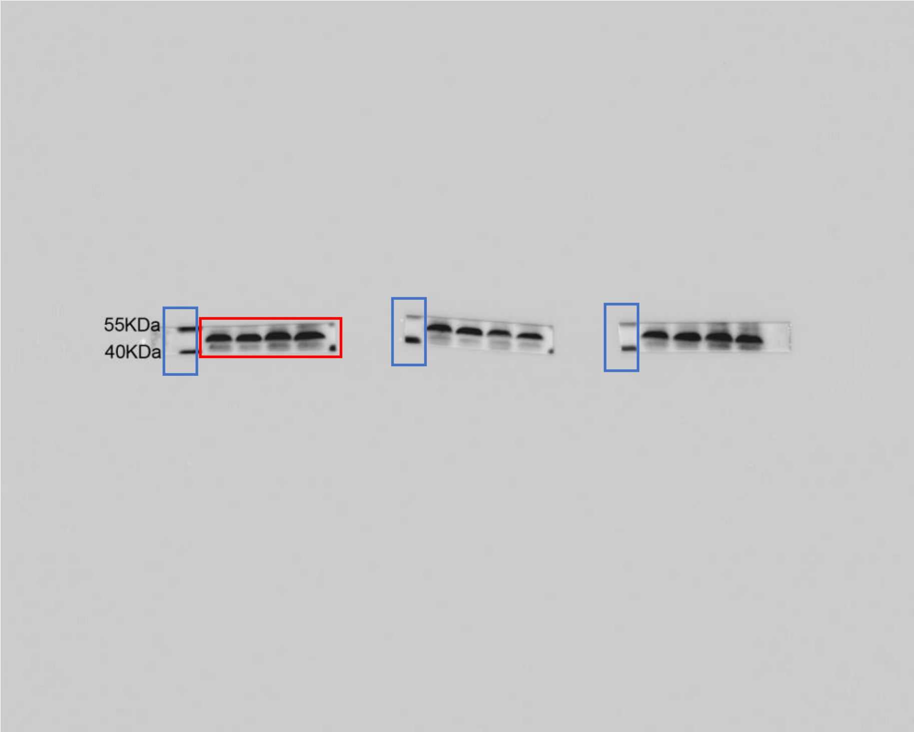

Original western blot images of β-actin; this band was partially cleaved for the detection of other proteins before hybridization with the antibody.

Figure3 D

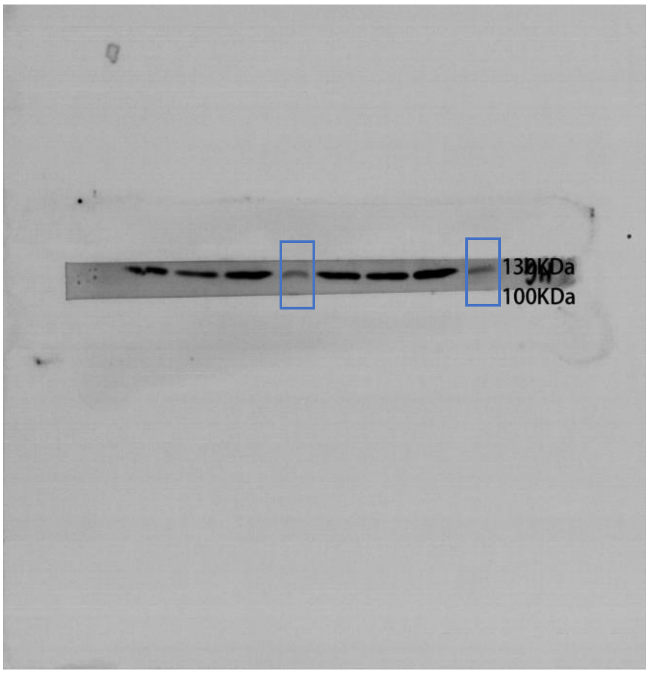

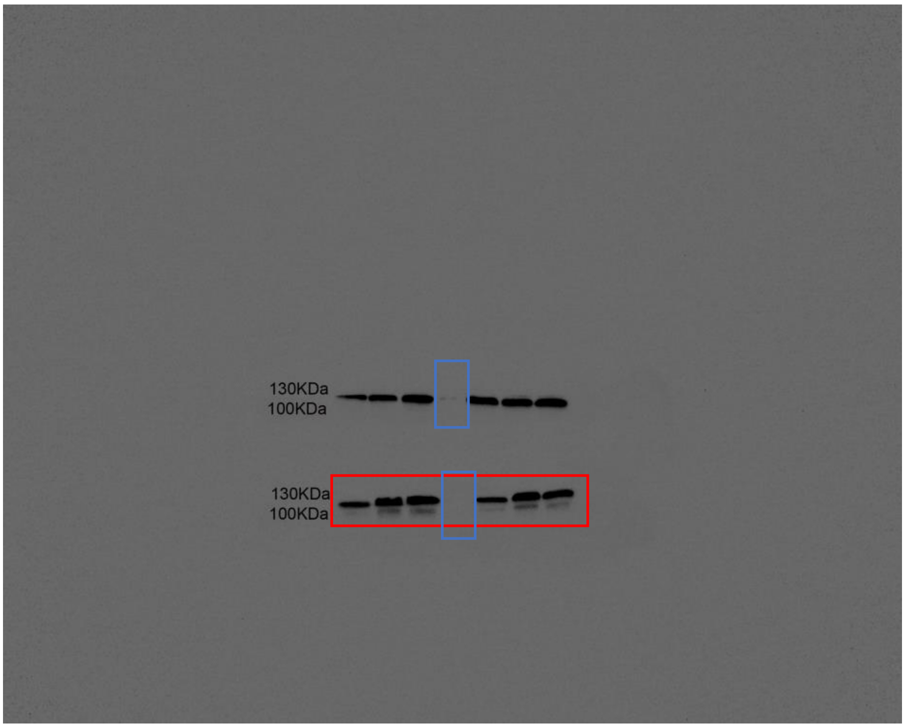

Original western blot image of E-cadherin; this band was partially cleaved for the detection of other proteins before hybridization with the antibody.

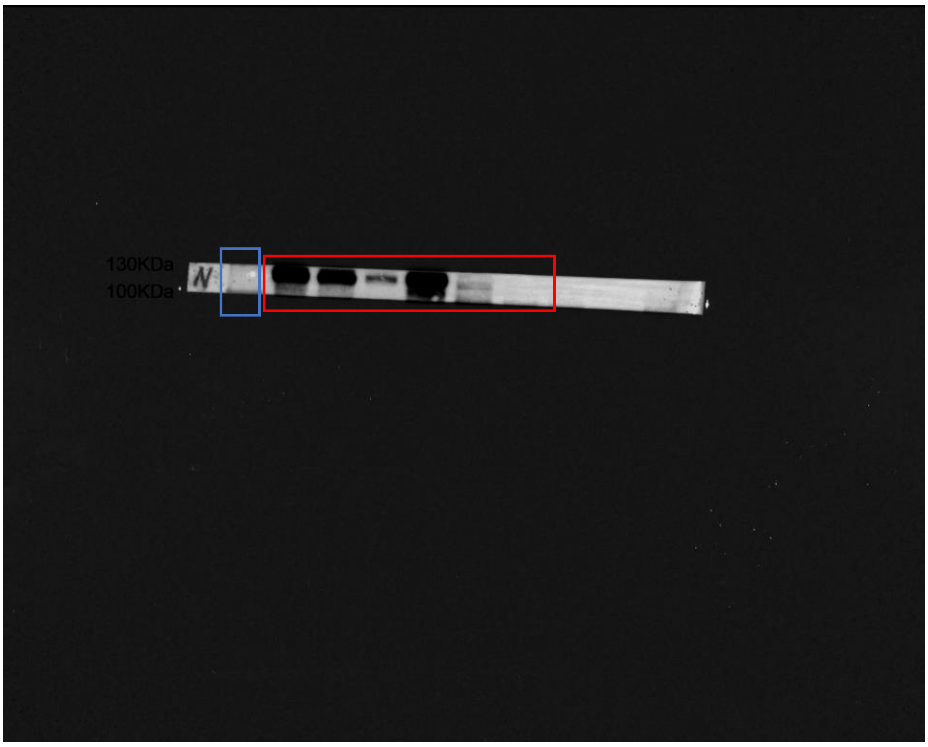

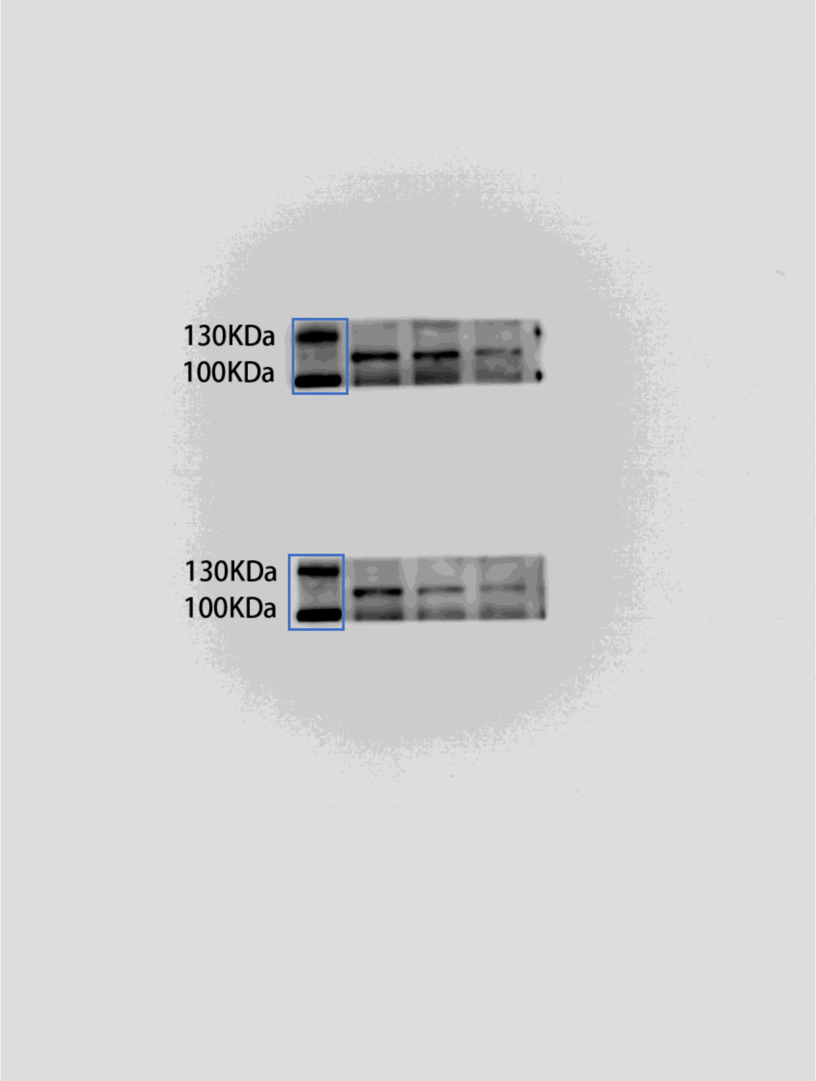

Original western blot image of N-cadherin; this band was partially cleaved for the detection of other proteins before hybridization with the antibody.

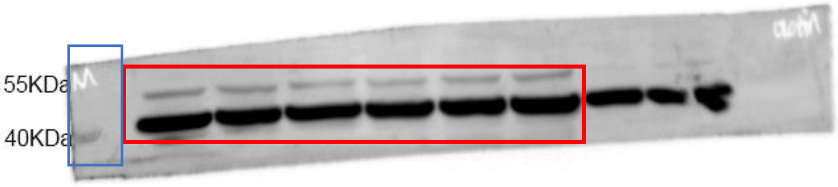

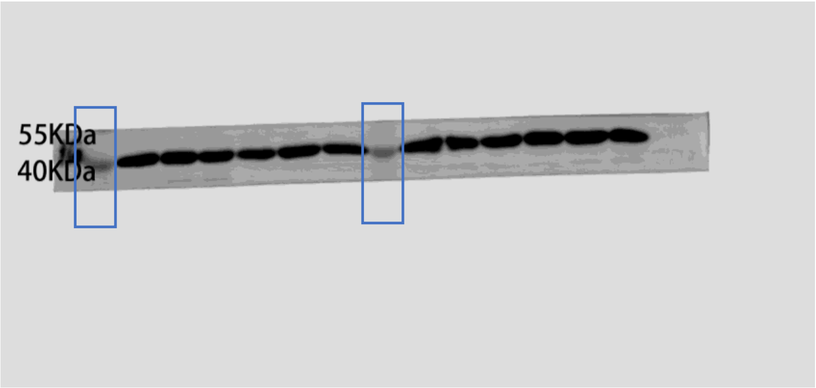

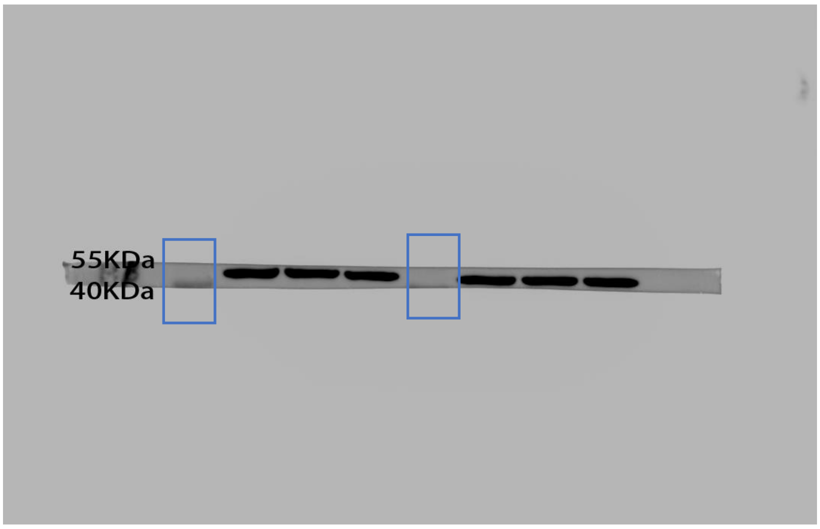

Original western blot images of β-actin; this band was partially cleaved for the detection of other proteins before hybridization with the antibody.

Figure4 E

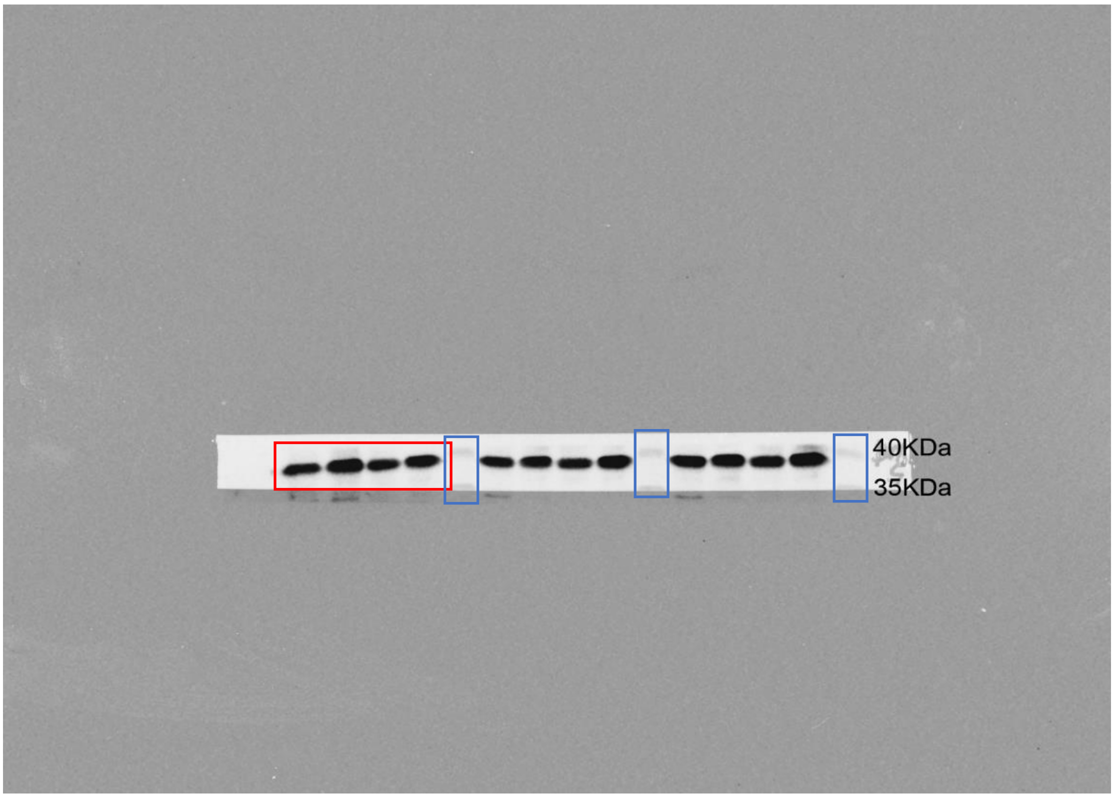

Original western blot images of Wnt3a(TOP2A-OE).

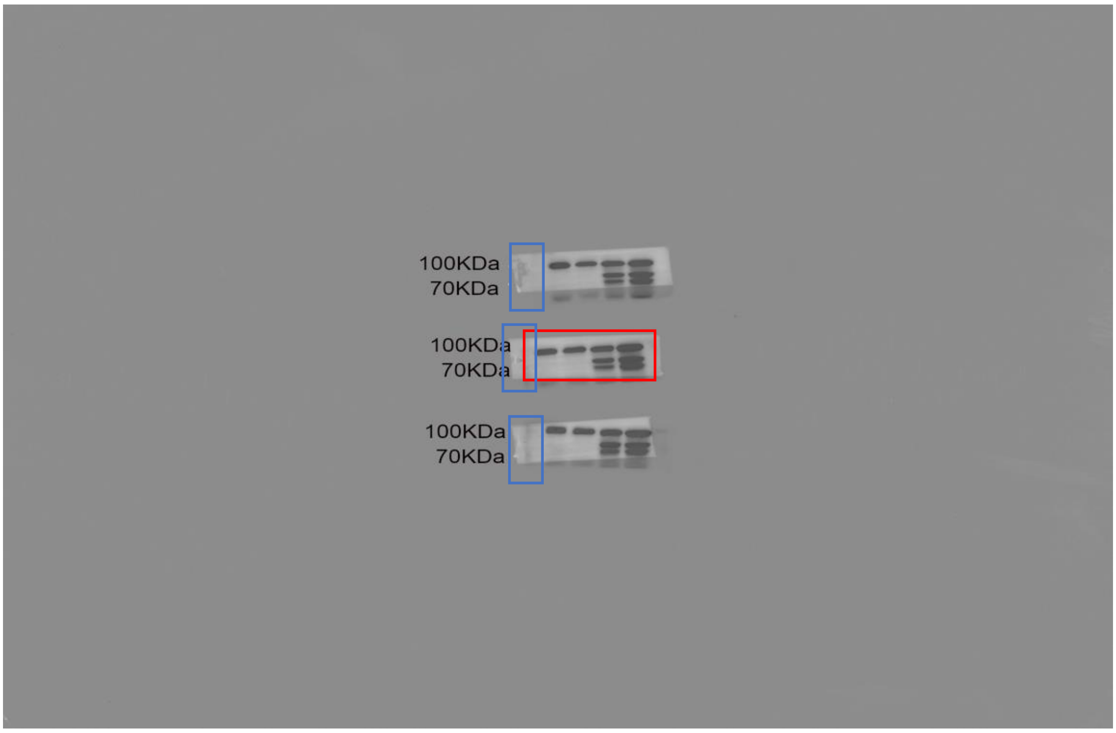

Original western blot image of β-catenin(TOP2A-OE) ; this band was partially cleaved for the detection of other proteins before hybridization with the antibody.

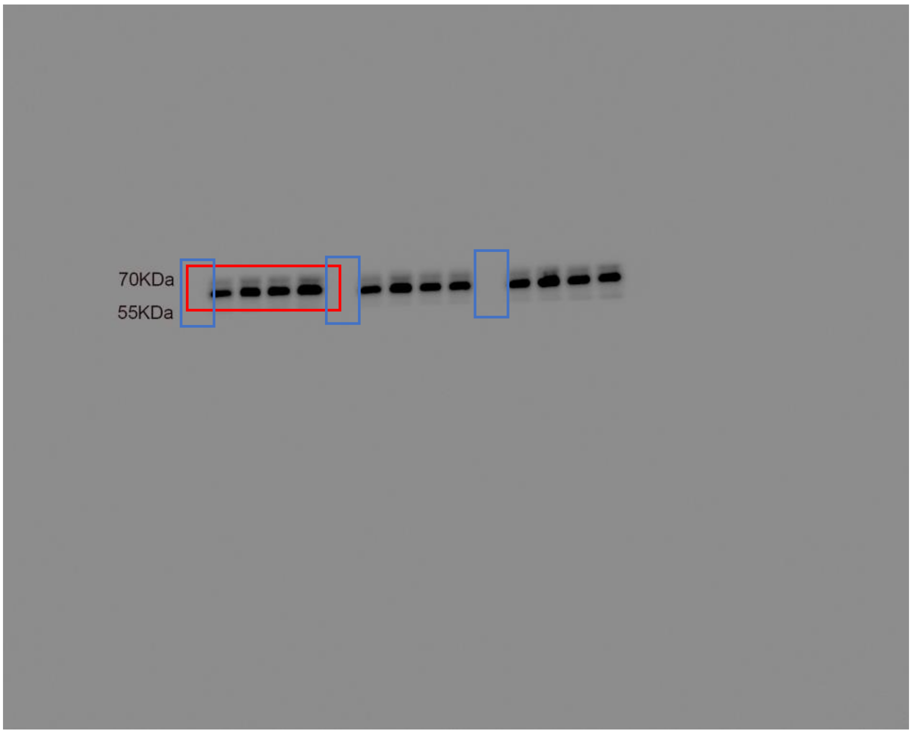

Original western blot image of c-Myc(TOP2A-OE).

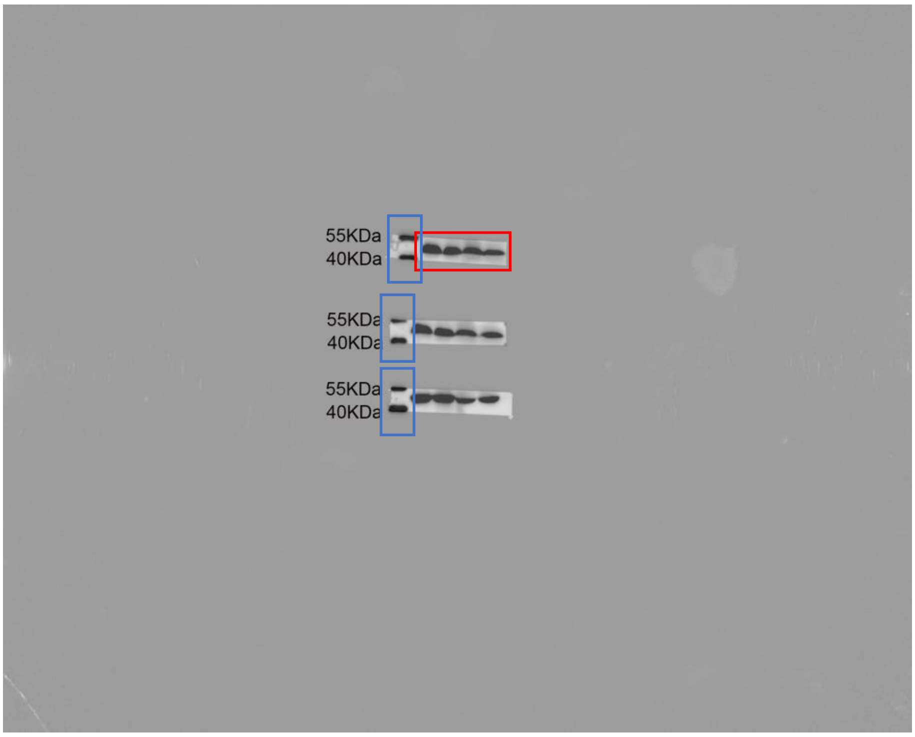

Original western blot images of β-actin(TOP2A-OE); this band was partially cleaved before hybridization with the antibody for detection of other proteins.

Figure4 E

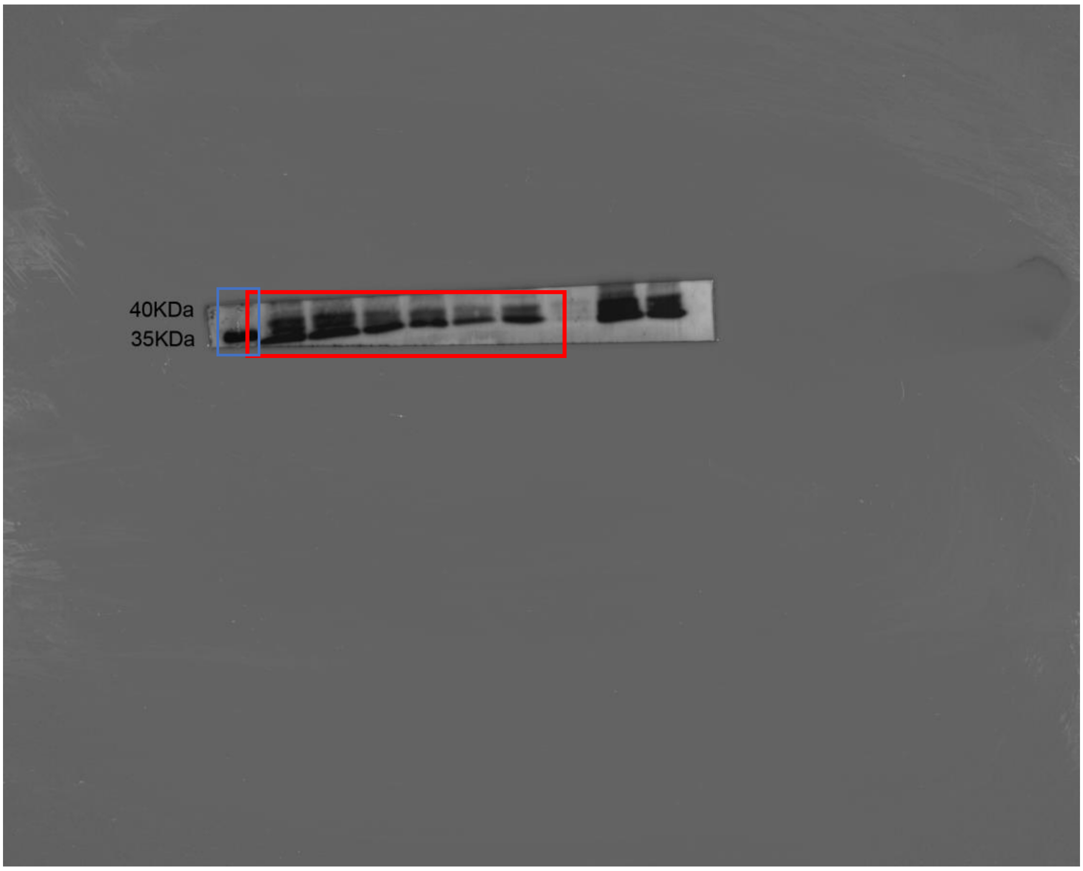

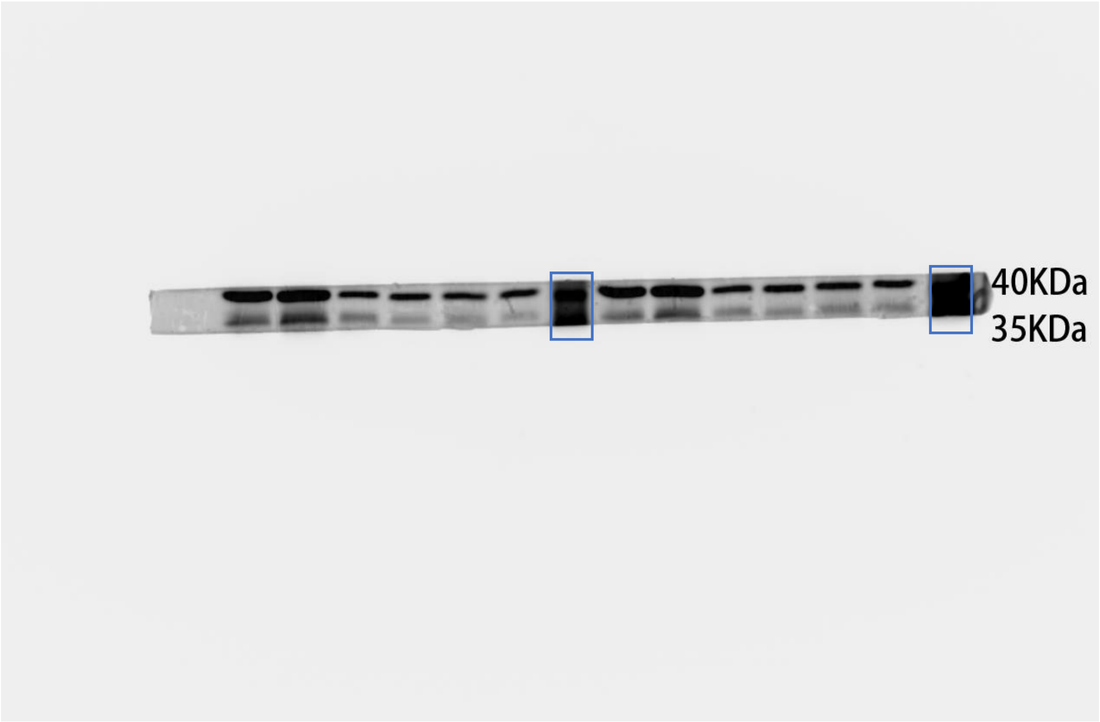

Original western blot images of Wnt3a(TOP2A-SiRNA).

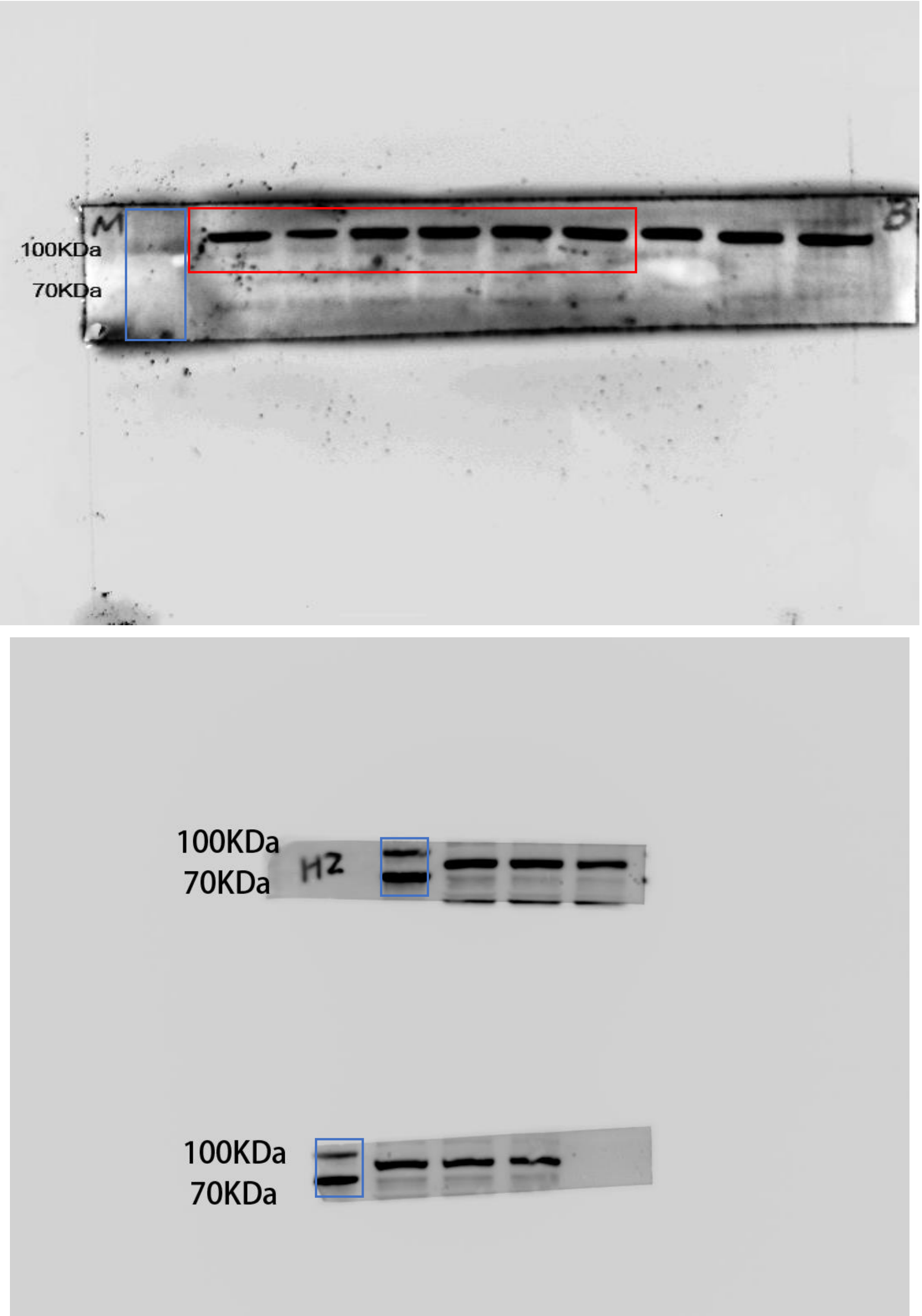

Original western blot image of β-catenin(TOP2A- SiRNA) ; this band was partially cleaved for the detection of other proteins before hybridization with the antibody.

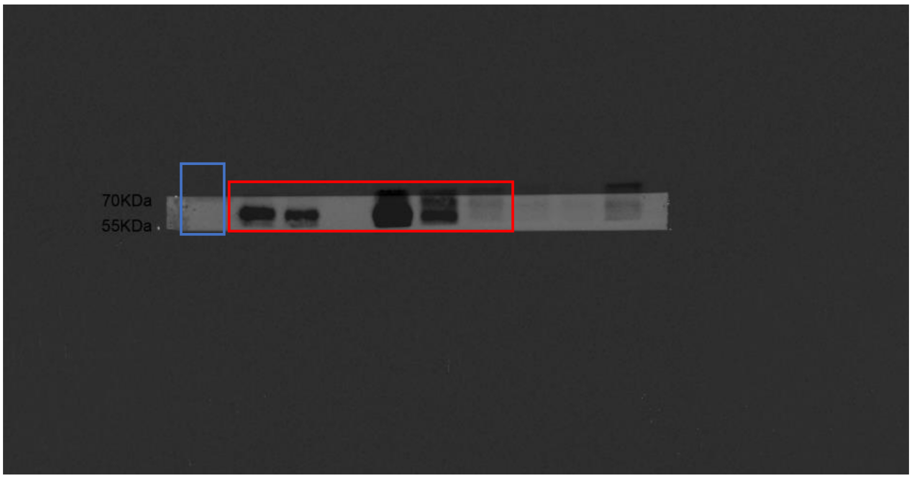

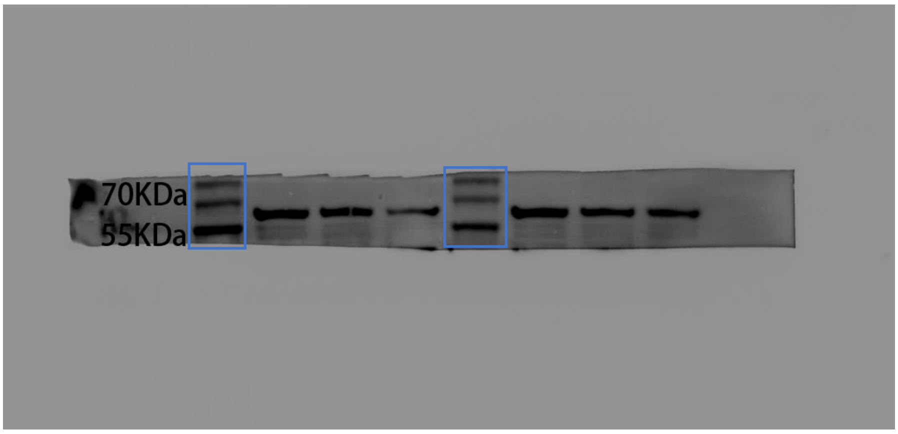

Original western blot image of c-Myc(TOP2A- SiRNA) ; this band was partially cleaved for the detection of other proteins before hybridization with the antibody.

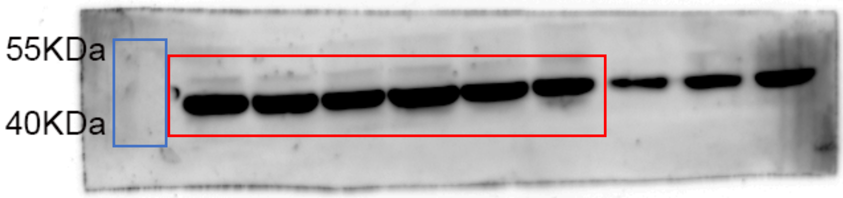
55KDa
40KDa

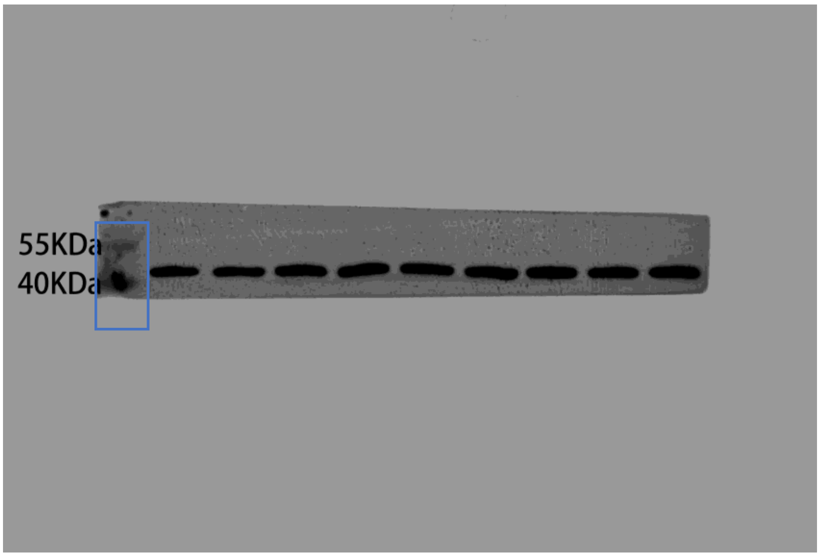
55KDa
40KDa

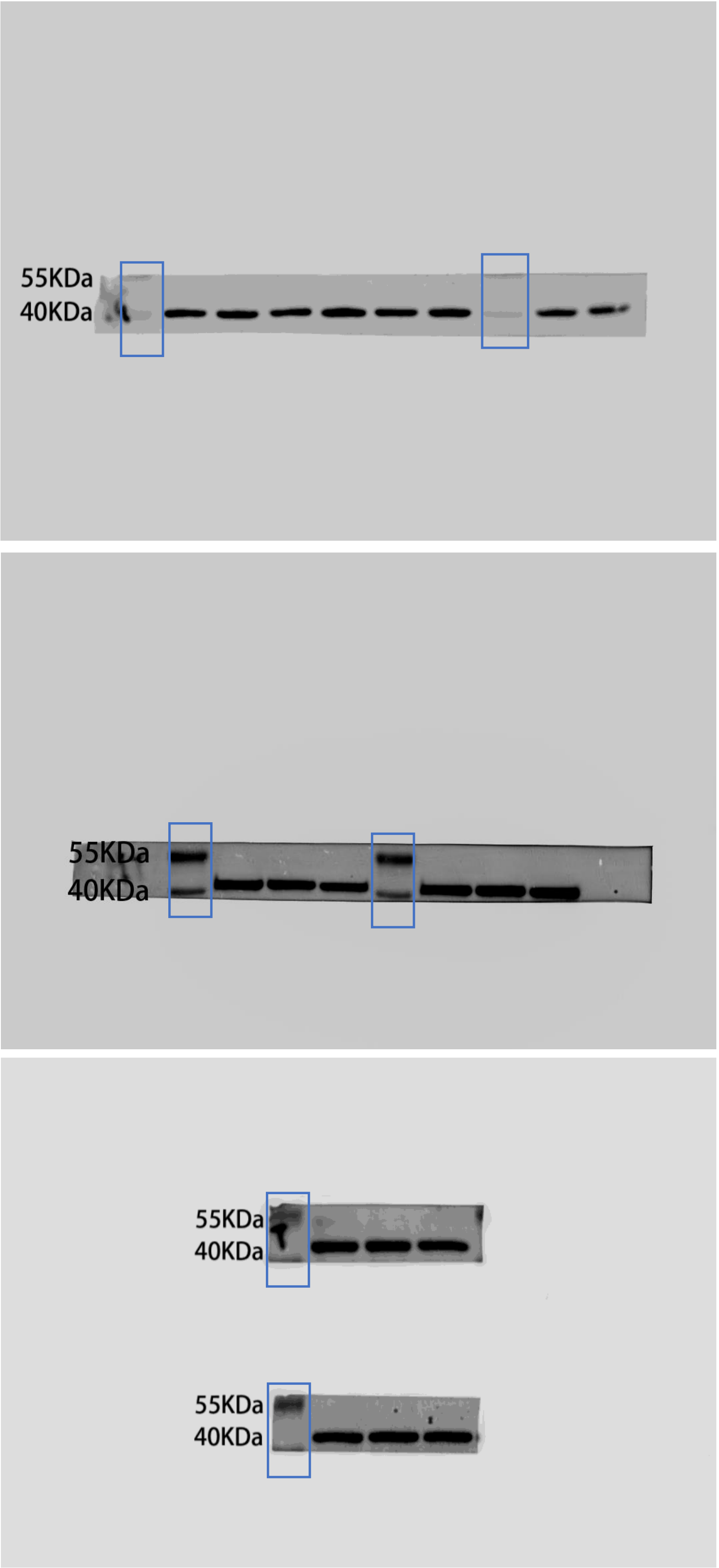

Original western blot images of β-actin(TOP2A- SiRNA); this band was partially

cleaved before hybridization with the antibody for detection of other proteins.

Figure5 B

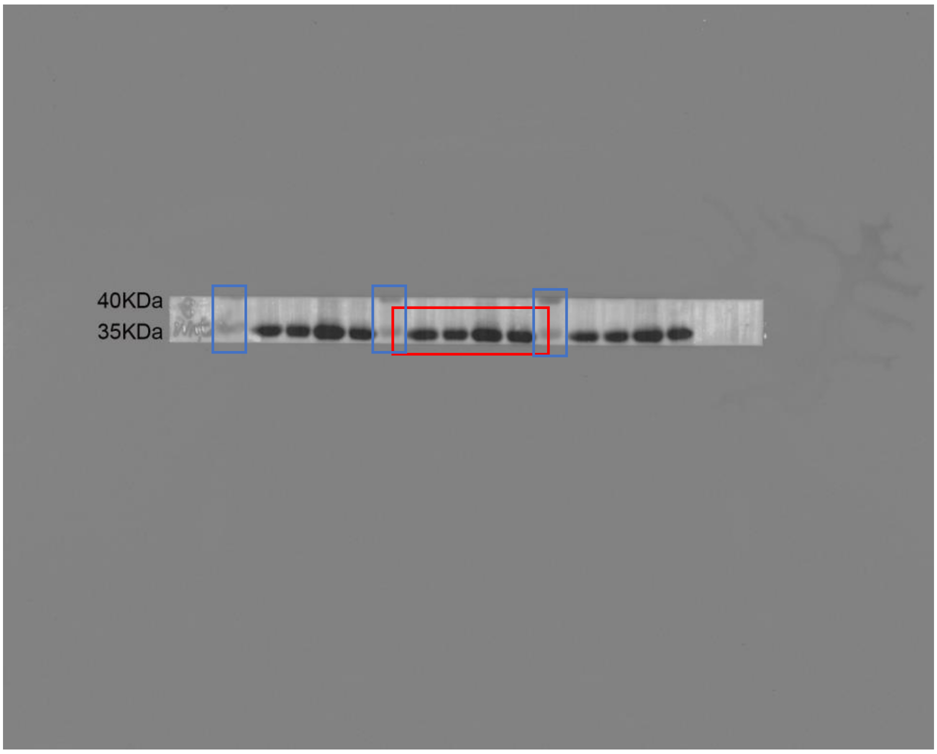

Original western blot images of Wnt3a; this band was partially cleaved for the detection of other proteins before hybridization with the antibody.

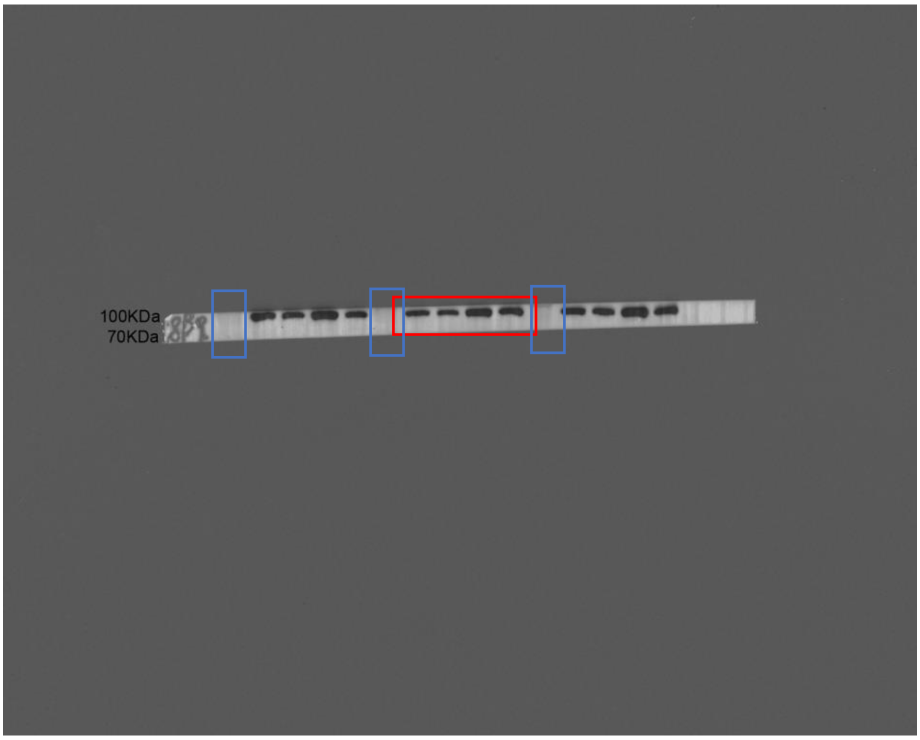

Original western blot image of β-catenin.

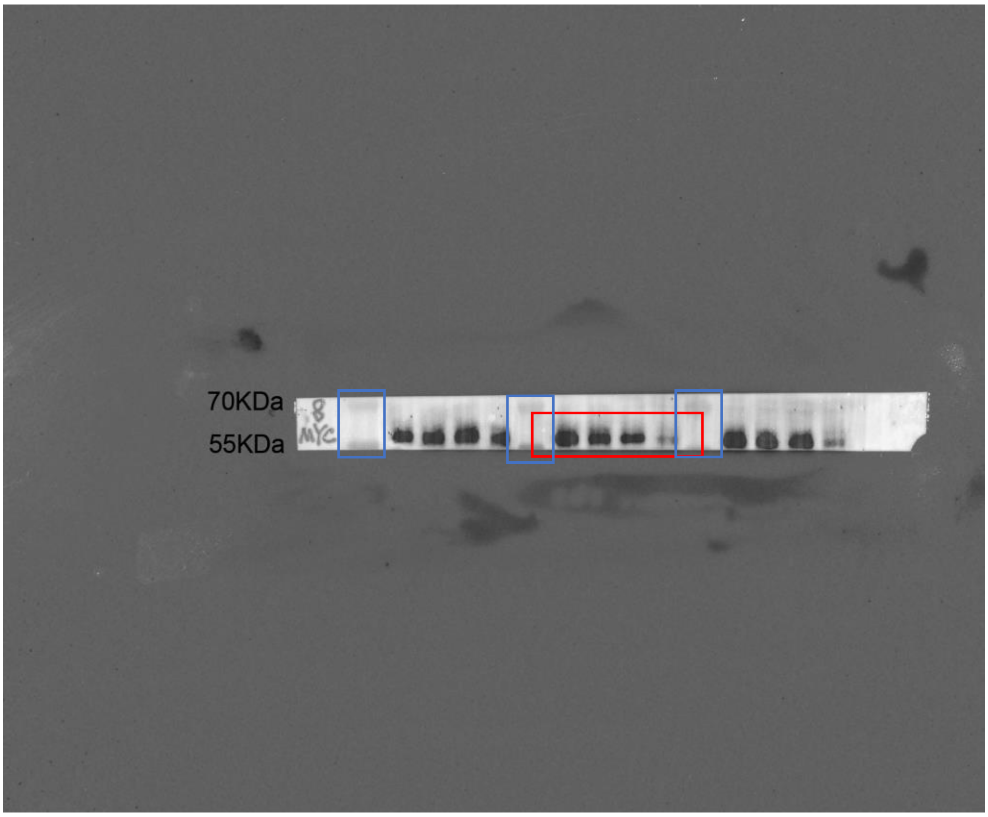

Original western blot image of c-Myc.

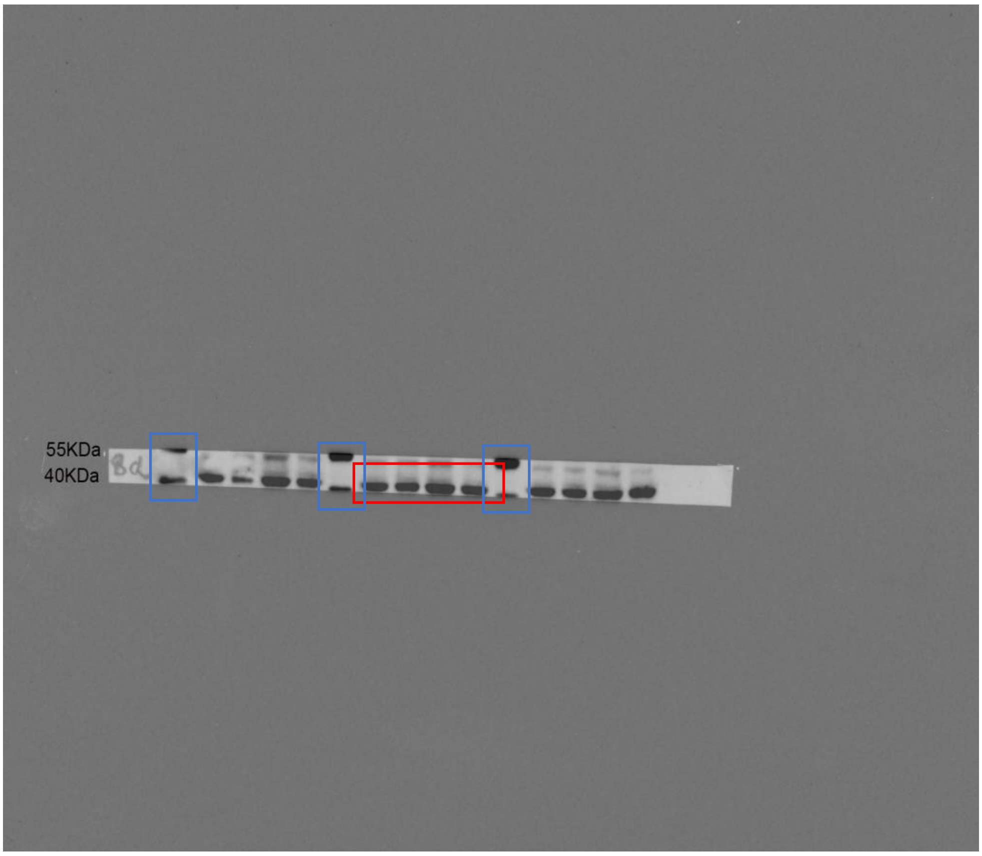

Original western blot image of β-actin.

Figure5 C

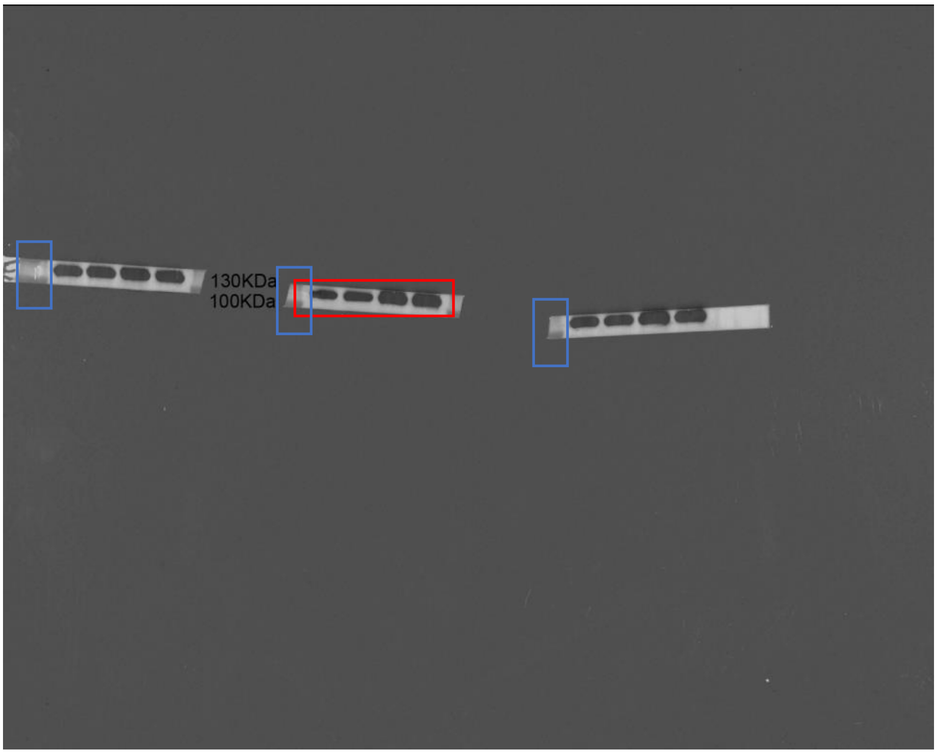

Original western blot images of E-cadherin; this band was partially cleaved for the detection of other proteins before hybridization with the antibody.

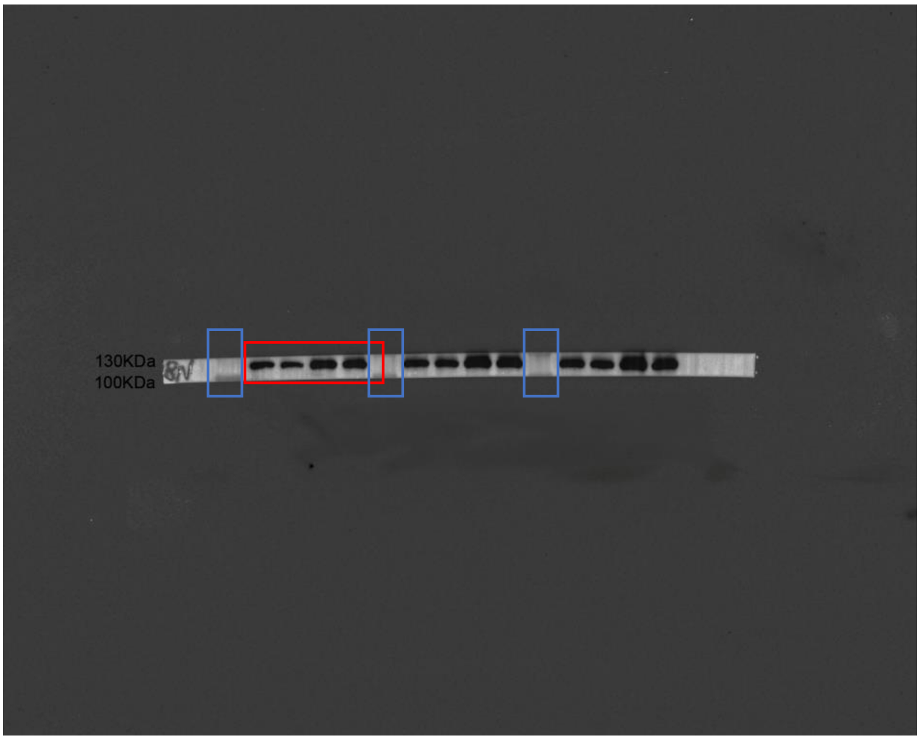

Original western blot image of N-cadherin.

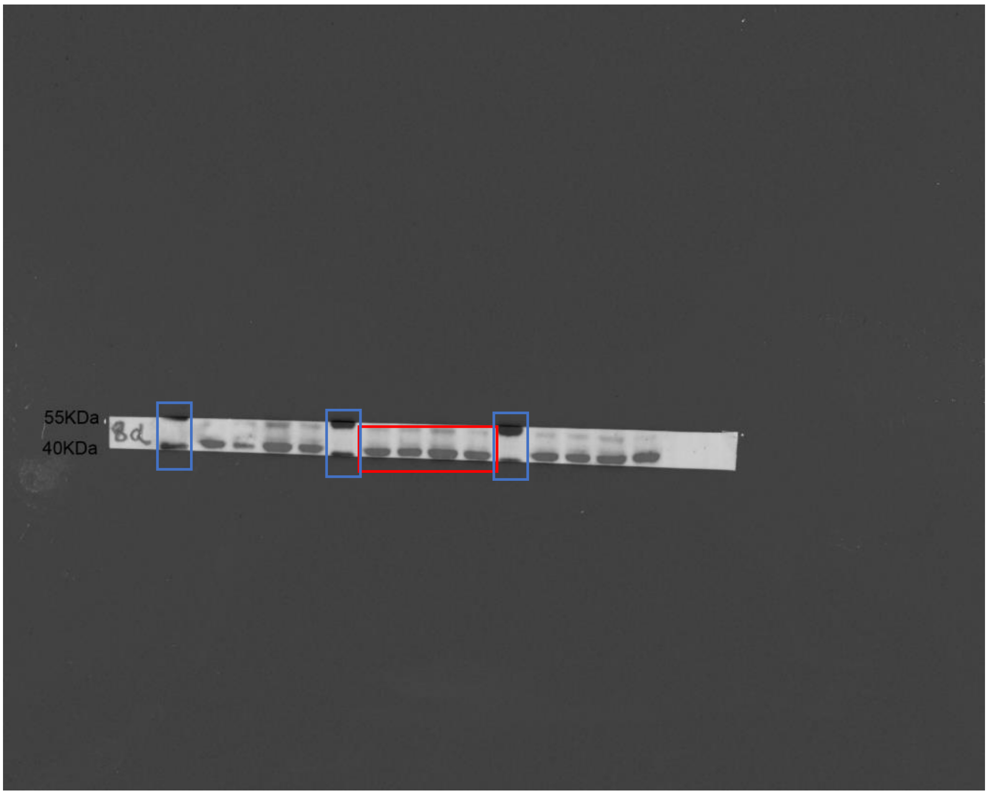

Original western blot image of β-actin.

Note: the red box indicates the area of the original blot used in the main image; the blue box indicates the hole where the protein mark is located. Most of the original strips provided were replicated three times in a single experiment.
